# Subphthalocyaninato Boron(III) Hydride: Synthesis, Structure and Reactivity

**DOI:** 10.1002/chem.202101991

**Published:** 2021-07-07

**Authors:** Lara Tejerina, Jorge Labella, Lara Martínez‐Fernández, Inés Corral, M. Victoria Martínez‐Díaz, Tomás Torres

**Affiliations:** ^1^ Department of Organic Chemistry Universidad Autónoma de Madrid Campus de Cantoblanco 28049 Madrid Spain; ^2^ Institute for Advanced Research in Chemical Sciences (IAdChem) Universidad Autónoma de Madrid Campus de Cantoblanco 28049 Madrid Spain; ^3^ IMDEA-Nanociencia c/Faraday 9, Campus de Cantoblanco 28049 Madrid Spain; ^4^ Department of Chemistry Universidad Autónoma de Madrid Campus de Cantoblanco 28049 Madrid Spain

**Keywords:** borenium catalysis, boron hydride, hydroboration, silylium catalysis, subphthalocyanine

## Abstract

Subphthalocyanine (SubPc) chemistry has been limited so far by their high sensitivity toward strong nucleophiles. In particular, the substitution of the axial chlorine atom by a nucleophilic group in the case of less‐reactive SubPcs, such as those bearing electron‐withdrawing peripheral substituents, presents some limitations and requires harsh conditions. By taking advantage of the electrophilic character of DIBAL‐H, it has been possible to prepare for the first time SubPc‐hydride derivatives that exhibit high reactivity as hydroboration reagents of aldehydes. This hydride transfer requires using a typical carbonyl activator (trimethylsilyl triflate) and only one equivalent of aldehyde, affording SubPcs with an axial benzyloxy group in good yield. This transformation has proven to be a useful alternative method for the axial functionalisation of dodecafluoroSubPc, a paradigmatic SubPc derivative, by using electrophiles for the first time. Considering the increasing interest in SubPcs as electron‐acceptor semiconductors with remarkable absorption in the visible range to replace fullerene in organic photovoltaic (OPV) devices, it is of the utmost importance to develop new synthetic methodologies for their axial functionalisation.

## Graphical abstract

**For the first time** subphthalocyanine‐hydride derivatives that exhibit high reactivity as hydroboration reagents of aldehydes have been prepared. This transformation has proven to be a useful alternative method for the axial functionalisation of dodecafluoro‐subphthalocyanines using electrophiles. Considering the increasing interest in SubPcs as electron‐acceptor semiconductors to replace fullerene in organic photovoltaic devices, it is of the utmost importance to develop new synthetic methodologies for their axial functionalisation.

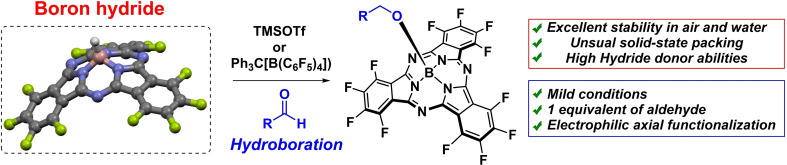

Subphthalocyanines (SubPcs) have been extensively investigated as active chromophores in different technological and biomedical applications, such as energy conversion or photodynamic therapy.[^1^, ^2^, ^3^] This unique nonplanar aromatic macrocycle, which is composed of three N‐fused 1,3‐diiminoisondoline units around a central tetrahedral boron atom and a fourth substituent that occupies the axial position, exhibits multiple exciting properties easily tailored by functionalisation. Whereas chemical modification of the substituents at the periphery leads to remarkable changes in the electronic properties of SubPcs, replacement of the axial ligand (typically a chlorine atom) mainly affects physical properties such as solubility, stability, or tendency towards aggregation.

Following this vein, the substitution reaction of the halogen atom by alcohols has been by far the most recurrent method for axial functionalisation of SubPc on account of the greater stability of the resulting alkoxylated derivatives.^[4]^ In particular, phenol derivatives have been widely employed to prepare a variety of SubPc‐based multicomponent systems with interesting properties.^[1]^ This reaction usually proceeds by heating the mixture of the SubPc and an excess of phenol in a high boiling point solvent, such as toluene or *o*‐dichlorobenzene (*o*‐DCB). Optionally, weak bases such as trialkylamines are added to neutralise the hydrogen chloride generated. Alternatively, the use of a Lewis acid, namely AlCl_3_, which binds the axial chlorine atom prior to the addition of the nucleophile, also facilitates the axial substitution reaction.^[5]^ Experimental evidences and theoretical calculations have suggested that these reactions evolve through the coordination of the axial ligand lone pairs to either an acidic proton or a Lewis acid, thereby weakening of the B−X bond, thus assisting the attack of nucleophiles to the now more electrophilic boron atom.^[6]^ Consistently with this mechanism, SubPcs bearing electron deficient peripheral substituents show lower axial reactivity, which is ascribed to their stronger B−X bond and, thus, requiring high reaction temperatures and longer reaction times, which may ultimately lead to SubPc decomposition. The use of more nucleophilic aliphatic alcohols is also precluded. In this case, an alternative synthetic strategy is recommended, based on the in‐situ generation of an activated SubPc bearing an axial triflate, allowing milder reaction conditions.^[7]^

More rarely, other carbon, nitrogen and sulfur‐based nucleophiles have been introduced as axial substituents using these and other methodologies. Up to now, however, the peculiar axial functionalisation with hydrogen has never been described in SubPcs. Given the electron‐donor capacity and the small size of the hydride substituent, this innovative derivative may possess a particular chemical reactivity and interesting electronic and aggregation properties. Thus, we have focused our research on the synthesis of such unprecedented SubPc derivatives.

The great ability of aluminium to coordinate the axial chlorine atom of SubPcs put forward diisobutylaluminum hydride (DIBAL‐H) as ideal reagent to carry out the axial replacement of chloride by hydride (Scheme 1), using a similar procedure to the one reported by Osuka et al. for the preparation of the subporphyrin (SubP) analogue.^[8]^ To our delight, upon optimisation, SubPc **1 a** reacted with five equivalents of DIBAL‐H in 3 minutes at −55 °C to yield SubPc hydride **2 a** in 49 % yield after purification by a short silica plug. Surprisingly, **2 a** showed an outstanding chemical stability, remaining unaltered to the air and in protic solvents as methanol or water. Adjustments only in the number of equivalents of DIBAL‐H allowed the preparation of other SubPcs hydrides, namely **2 b** and **2 c**, bearing iodine atoms at different peripheral positions. Unfortunately, owing to the poor solubility of the starting SubPc **1 d** under the reaction conditions, only very low yield of **2 d** was obtained (for more details, see the Supporting Information).

**Scheme 1:**
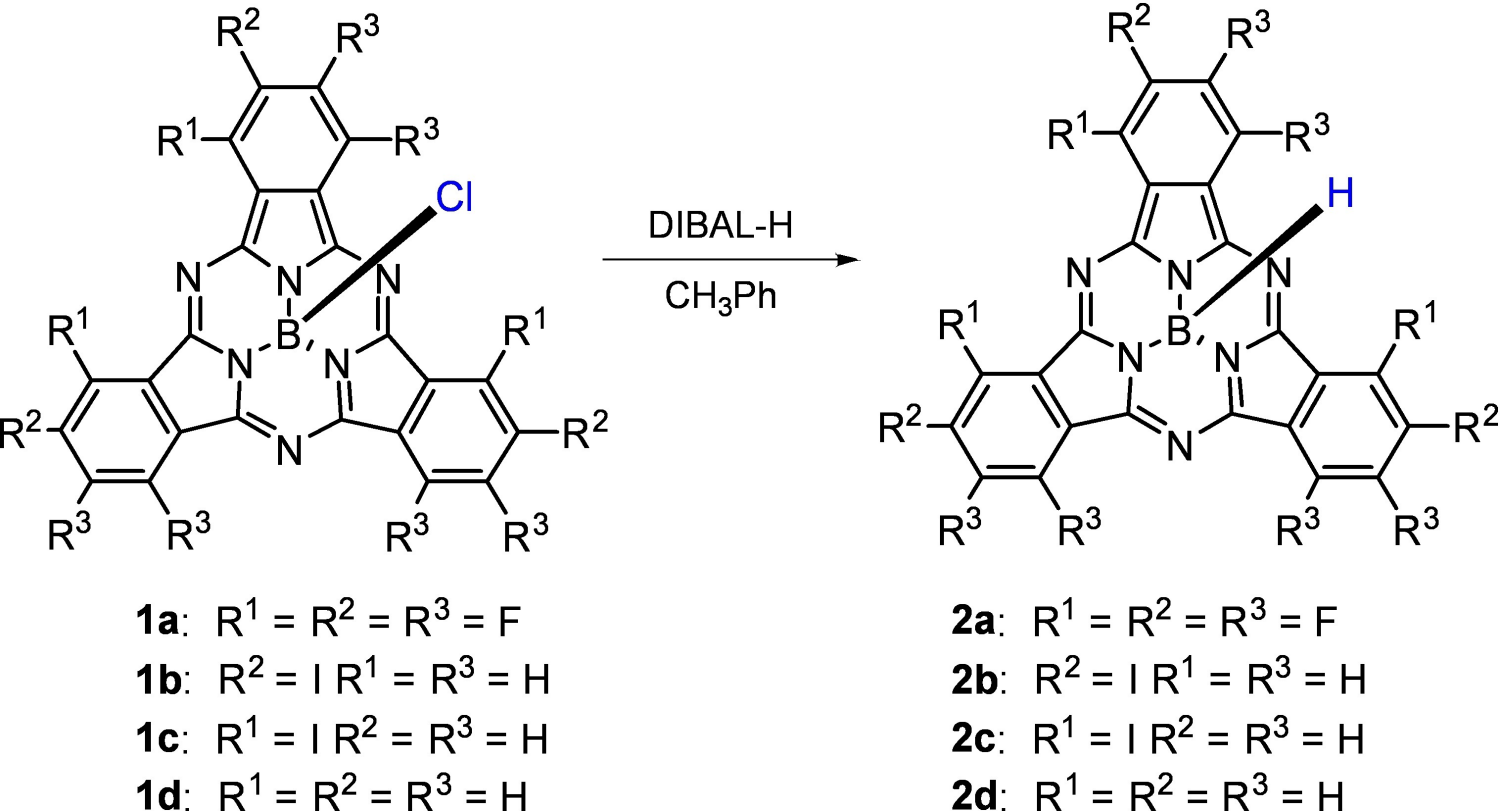
Synthesis of subphthalocyanine hydrides **2**.

Unequivocal spectroscopic characterisation of these new members of the SubPc family was carried out by NMR, UV/Vis, FTIR spectroscopies, MS, and HRMS. Furthermore, the structure of SubPcs **2 a** and **2 d** was confirmed by X‐ray diffraction analysis. The ^1^H NMR spectra of SubPcs **2** in CDCl_3_ showed a characteristic B−H signal as a broad doublet around −0.90 ppm with *J*_BH_=155 Hz at 55 °C. The broadness produced by the rapid quadrupole induced relaxation of the ^11^B nucleus hinders the visualisation of the real quartet.^[9]^ The ^11^B NMR spectra of SubPcs **2** at 55 °C exhibit a broad doublet centred around −19.0 ppm, which converts into singlet in ^1^H‐decoupled ^11^B NMR spectrum. The ^11^B chemical shift is more than 4 ppm shielded with respect to starting SubPcs **1** (ca. −14.07 ppm) as a result of the more electron‐donating character of hydride substituent. The infrared spectra of SubPcs **2** display a B−H stretching vibration at ≈2480 cm^−1^, significantly greater than that reported for other SubP boron hydride derivatives (i. e., 2280 cm^−1^),^[8]^ thus suggesting a remarkably stronger B−H bond in ours. This result is in line with the exceptional stability of these SubPc hydrides.

Single crystals of **2 a** and **2 d** suitable for X‐ray analysis were obtained by slow evaporation of CHCl_3_ solutions (Figure 1, see the Supporting Information for further details). The B−H bond lengths were determined to be 1.208 and 1.167 Å in **2 a** and **2 d**, respectively. These B−H lengths are slightly shorter than those of previously reported SubP analogues (i. e., 1.257–1.266 Å), which once again suggests a stronger B−H bond in our SubPcs, in agreement with IR data. The X‐ray structures of **2 a** and **2 d** show the characteristic bowl‐shape of SubPcs, which was quantified measuring the distance between the boron atom and the plane defined by the three pyrrole nitrogen atoms (BDpyr), the three imine nitrogen atoms (BDimine) or the six outer terminal carbon atoms (BDterm).^[10]^ The BDpyr, BDimine, BDterm values are 0.663, 1.221, 2.677 Å for **2 a** and 0.632, 1.203, 2.681 Å for **2 d**, respectively. Interestingly, these values are significantly larger than those observed in the starting Cl‐SubPcs **1 a** and **1 d** ^[10]^ (0.589, 1.153, 2.583 Å for **1 a** and 0.583, 1.135, 2.472 Å for **1 d**), thus suggesting an increased sp^3^ character of the boron atom in **2 a** and **2 d**, because of the electron‐donating character of the axial hydride. The SubPc hydrides **2 a** and **2 d** show a remarkable different molecular packing, in line with previous reports wherein it was found that variations in the peripheral substituents (from hydrogen to fluorine) have a profound effect on the solid‐state arrangement of SubPcs.^[10]^ However, notable unique changes are observed when introducing the axial hydride substituent, with respect to the corresponding axial halogen counterparts. Thus, while columnar packing is the favoured arrangement of Cl‐ or F‐F_12_SubPc,^[12]^ **2 a** is organized forming concave‐convex head‐to‐tail pairs by F⋅⋅⋅π, N⋅⋅⋅π and C⋅⋅⋅π interactions (see Figure S2 in the Supporting Information). More interestingly, the crystal structure of **2 d** is dominated by B−H⋅⋅⋅B interactions which produce a perfect co‐axial arrangement of macrocycles wherein the aromatic units are totally aligned. Such interactions give rise to a parallel columnar‐based organisation that, to the best of our knowledge, has no precedent in other SubPc derivatives (Figure S13).

**Figure 1:**
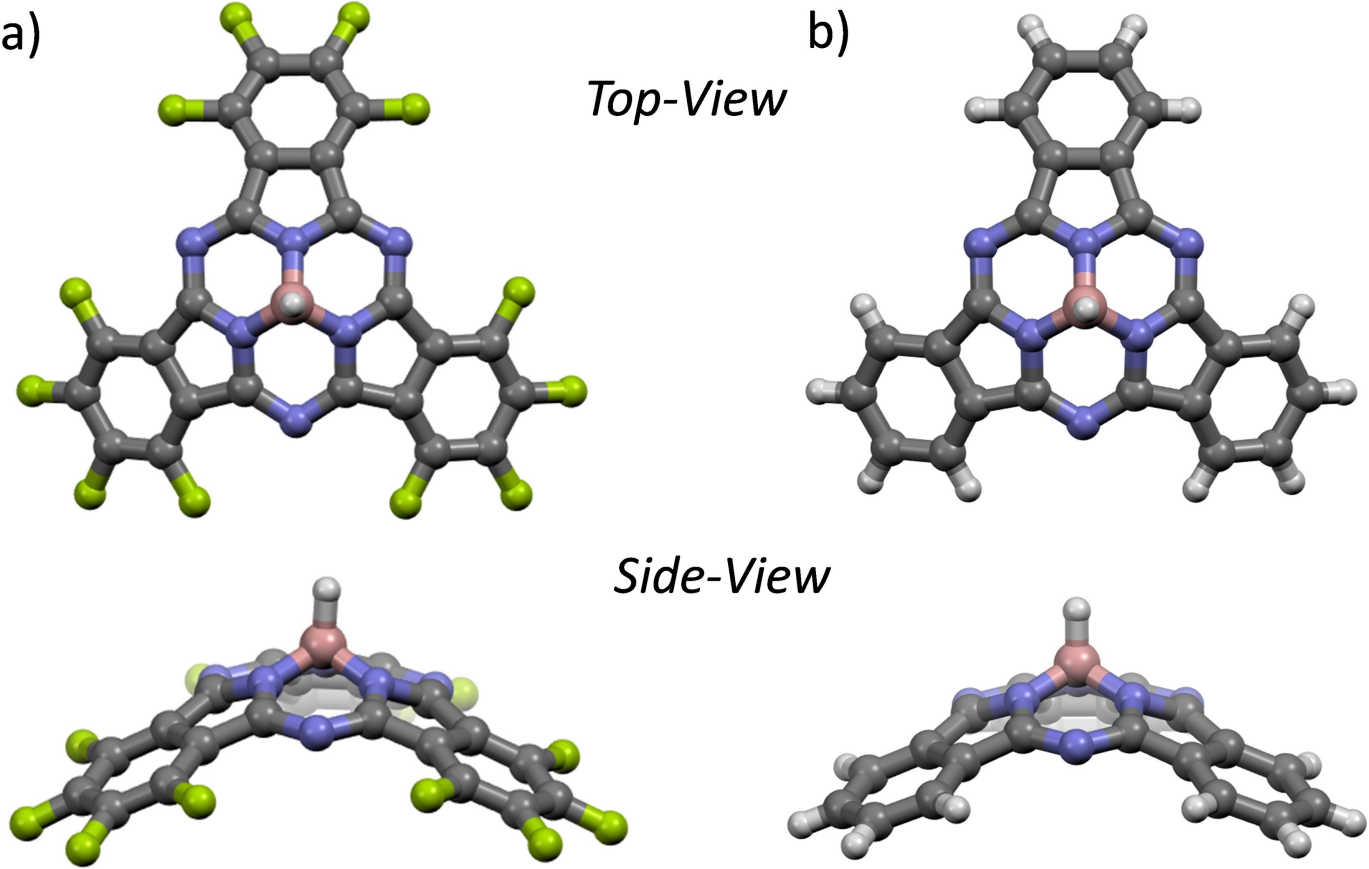
X‐ray crystal structures of a) SubPc **2 a** and b) SubPc **2 d**. Colour code: carbon in grey, fluorine in lime, hydrogen in white, nitrogen in blue and boron in pink.

The good yield of formation and great stability of SubPc‐hydride **2 a** encouraged us to explore a new methodology for the axial functionalisation of SubPcs through an unprecedented approximation that implies an electrophilic reagent, instead of commonly used nucleophiles, and hence avoiding macrocycle decomposition. For this purpose, the potential of SubPc **2 a** as hydroboration reagent of aromatic aldehydes was initially evaluated (Scheme 2).

**Scheme 2:**
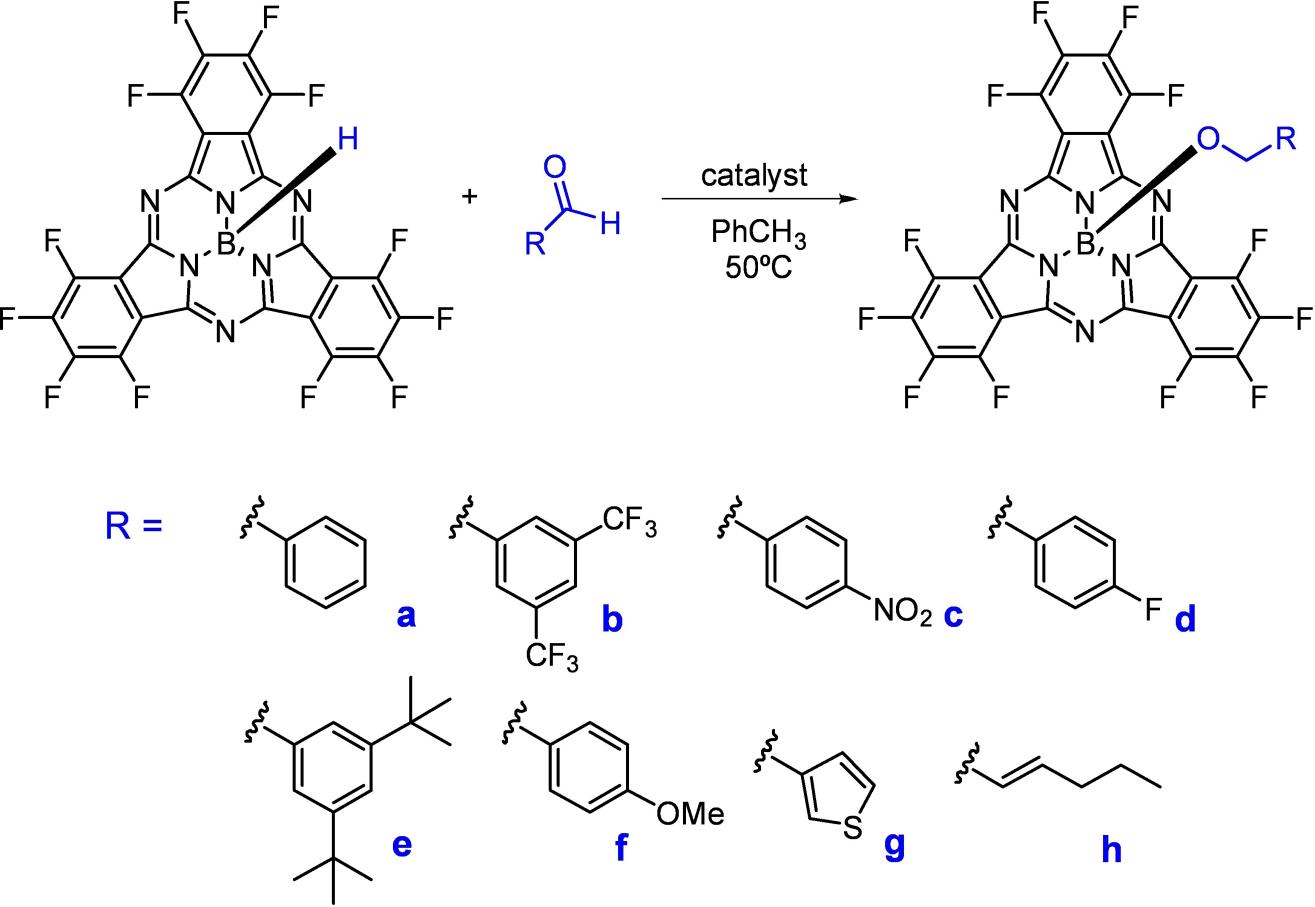
Catalysed hydroboration of aldehydes by SubPc **2 a**.

First, **2 a** was reacted with one equivalent of benzaldehyde (**a**) in toluene at 100 °C without success (Table 1, entry 1). Interestingly, the addition of a catalytic amount (10 %) of a carbonyl‐activator such as TMSOTf led to the desired hydroboration product,^[13]^ SubPc **3 a**, in excellent yield after 20 min at 50 °C in toluene (Table 1, entry 2). Moreover, the catalysis mediated by the borenium cation SubPcB^+^ generated in situ from 10 % Ph_3_C[B(C_6_F_5_)_4_]) was also tested,^[8]^ obtaining **3 a** with similar yield but longer reaction time (Table 1, entry 10 and Scheme 2, see Supporting Information for mechanistic details). Taking the excellent activity of TMSOTf into account, the structural scope of this hydroboration of aldehydes with **2 a** was studied employing aromatic aldehydes **a**–**h** with different electronic features (Table 1).

**Table 1:** Structural scope for the catalysed hydroboration of aldehydes by SubPc **2 a**.

	R	*t* [min]	SubPc	Yield [%]^[a]^
1^[d]^	**a**	>1440	**3 a**	–^[b]^
2^[c]^	**a**	20	**3 a**	93
3^[c]^	**b**	180	**3 b**	73
4^[c]^	**c**	480	**3 c**	81
5^[c]^	**d**	120	**3 d**	74
6^[c]^	**e**	30	**3 e**	89
7^[c]^	**f**	–	**3 f**	–^[b]^
8^[c]^	**g**	–	**3 g**	–^[b]^
9^[c]^	**h**	60	**3 h**	69
10^[e]^	**a**	80	**3 a**	92
11^[e]^	**f**	60	**3 f**	67
12^[e]^	**g**	100	**3 g**	75

[a] Yields were determined after purification by column chromatography. [b] Undetected. [c] A 10 % mol of TMSOTf was employed as catalyst. [d] without catalyst, in toluene at 100 °C. [e] A 10 % of Ph_3_C[B(C_6_F_5_)_4_] was employed to generate the SubPcB^+^ catalyst.

From these results a clear trend was observed, being the reaction time significantly longer for electron‐poor aldehydes (entries 3 and 4) in comparison with the model substrate **2a** (entry 2). Notably, when aromatic aldehydes bearing strong electron‐donating groups (namely, 4‐methoxybenzaldehyde and thiophene‐carbaldehyde, entries 7 and 8) were employed, the corresponding SubPcs **3 f** and **3 g** were not obtained. In these cases, upon disappearance of the initial SubPc **2 a** in the TLC plate after 10 min of reaction, the formation of new pink TLC spots (SubPc side products) were detected. These SubPc by‐products were identified as SubPcs **4**, **5**, and hydroxy‐SubPc **6** (see Supporting Information), probably originated from nucleophilic attack to the benzylic position of the axial substituent of the Lewis acid‐base adduct formed between the catalyst (TMSOTf) and the final alkoxy SubPc **3** product (Scheme 3). Indeed, when SubPc **3 f** was subjected to reaction with 10 % mol of the TMSOTf catalyst under the reaction conditions, an immediate transformation to give a mixture of SubPcs **4**, **5**, and **6** was observed (see the Supporting Information). Fortunately, the reduction of these electron‐rich aldehydes **f** and **g** was achieved by the alternative borenium catalysis in good yields (Table 1, entries 11 and 12).

**Scheme 3:**
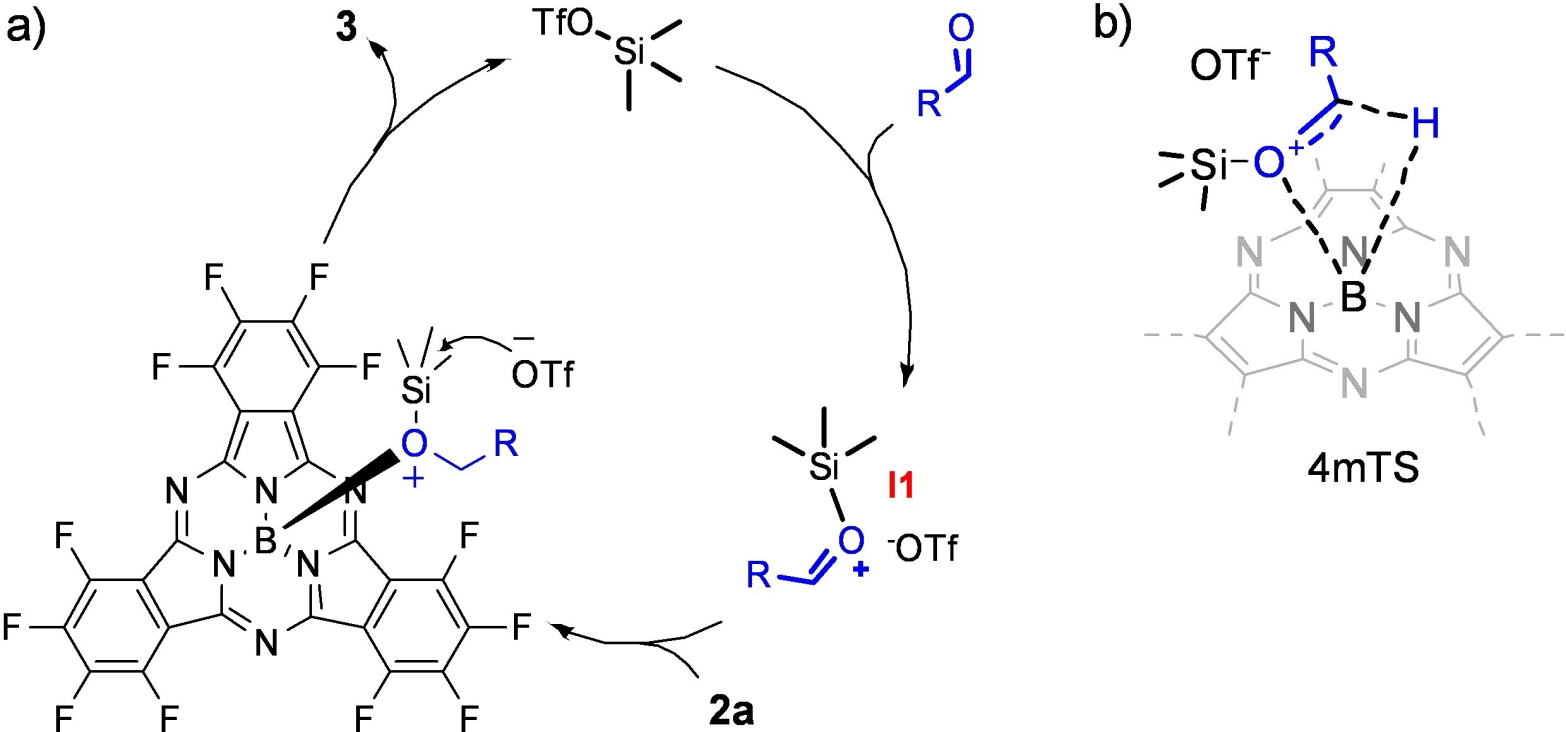
a) Alternative concerted reaction pathway for the hydroboration of aldehydes by **2 a** under TMSOTf catalysis. b) Proposed four‐member transition state (4mTS) for the hydride transfer step.

Finally, the mechanism of the hydroboration reaction of aldehydes by SubPc **2 a** under TMSOTf catalysis was explored. A first plausible catalytic cycle was proposed (Scheme S3). This mechanism would imply a transmetallation step between the silyl ether resulting from the reduction of the activated aldehyde and the in situ formed triflate‐SubPc. However, since it has been demonstrated that this transmetallation step does not occur (see the Supporting Information for more details), it can be inferred that triflate‐SubPc is not formed during the reaction, thus the hydride transfer step must follow other reaction pathway. Taking into account previous experimental and theoretical investigations about axial functionalisation of SubPcs derivatives,[^5^, ^13^] a second mechanistic hypothesis was postulated (Scheme 3a), in which the hydride transfer would proceed through a four‐member transition sate (4mTS; Scheme 3b) wherein the C−H and B−O bonds are simultaneously formed.

To shed light on the hydride transfer mechanism, the reaction pathway was investigated at molecular level by means of density functional theory (see the Supporting Information for further details). Figure 2 depicts the potential energy profile for the model reaction.

**Figure 2:**
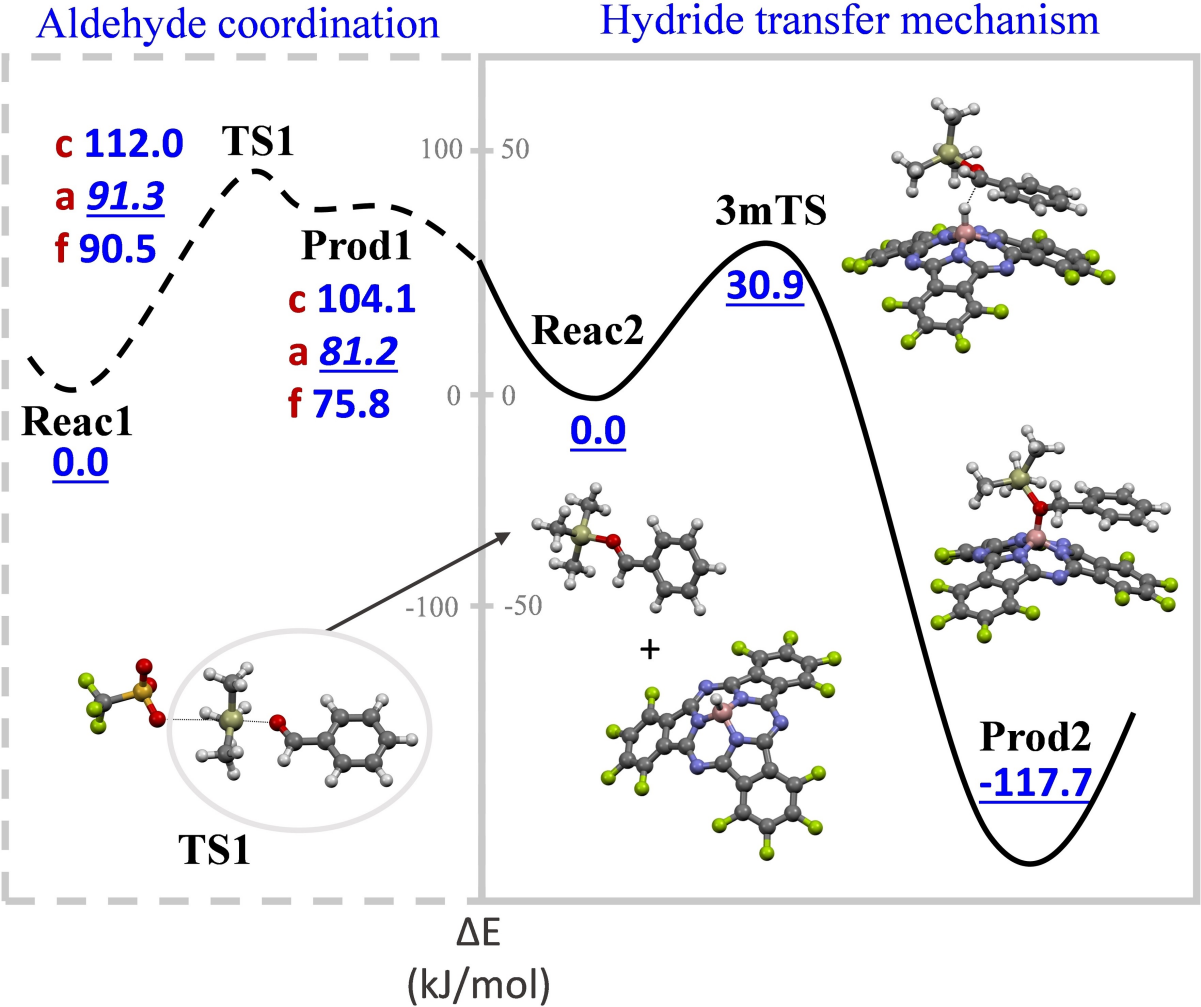
Potential energy profile for the activation of the aldehyde, and the hydride transfer step for the hydroboration of benzaldehyde by **2 a** catalysed by TMSOTf. Relative potential energy [kJ mol^−1^] vs. intrinsic reaction coordinates. Colour code: carbon in grey, fluorine in lime, hydrogen in white, oxygen in red, nitrogen in blue, silicon in pale green, sulfur in yellow, and boron in pink.

Interestingly, all the attempts performed to optimise the 4mTS (Scheme 3b) led to a more stable three‐membered transition state, 3mTS, which corresponds to the hydride exchange between boron and carbon atoms. The optimisation of 4mTS was only possible under structural constraints and, indeed, it was found to be ∼100 kJ mol^−1^ less stable than the 3mTS. These findings point, thereby, to the 3mTS as responsible for the energy barrier (30.9 kJ mol^−1^) connecting reactants and products, and being then, the main reaction pathway (see the Supporting Information for further discussion about the reaction mechanism).

Once the hydride transfer process was elucidated, the coordination of the TMSOTf to the aldehyde was studied also from the computational point of view (Figure 2, left). The energy barrier for this process was calculated to be 91.3 kJ mol^−1^, that is, significantly larger compared to the 3mTS, which implies that the aldehyde activation is the rate‐determining step. Thus, due to its key importance, the electronic effect of the substituent in the starting aldehyde was quantitatively evaluated. In agreement with the experimental results (Table 1), the strong influence of the aldehyde substituent in the energy barrier was confirmed by our calculations, being the coordination of aldehyde **f** (bearing an −OMe group) more favourable (78.2 kJ mol^−1^) than that of aldehyde **c** (−NO_2_ substituent, 112.0 kJ mol^−1^). This trend can be easily explained in terms of the decreased nucleophilicity of the aldehyde when electron withdrawing groups are attached to the benzene ring as substituents.

In conclusion, a general method for the synthesis of subphthalocyanine boron(III) hydrides (H‐SubPcs), so far unavailable, has been described. These H‐SubPcs exhibit an outstanding stability in air and protic solvents. Highly interesting is that perfluorinated SubPc‐H **2 a** was successfully employed as an efficient hydroboration agent of aldehydes under trimethylsilyl triflate catalysis, leading to alkoxy‐SubPcs **3** in good yield after short reaction times. This reaction has proven to be sensitive to the electronic features of the aldehydes, being more reactive those bearing an electron‐rich substituent. Moreover, the reaction mechanism for aldehyde activation and hydroboration of the aldehyde has been scrutinised at the molecular level by means of density functional theory. Surprisingly, unlike other axial substitution reactions of SubPc previously studied which are mediated by a four‐centre transition state (4mTS),[^6^, ^14^] in this case a three‐centre transition state (3mTS) is the most plausible one. Alternatively, other hydroboration conditions, such as borenium catalysis, also led to excellent results. These results open up the development of a new synthetic method for the axial functionalisation of electron‐poor SubPcs using electrophilic reagents. This method offers several advantages compared with previous reported methods, which require harsh conditions and excess of nucleophiles, leading to a high degree of macrocycle decomposition. The increasing interest in SubPcs as electron‐acceptor chromophores to replace fullerene in OPV devices heightens the interest in developing new synthetic methodology for their axial functionalisation. Further studies on the reactivity and properties of these unusual SubPc boron hydrides are underway in our laboratories.

Deposition numbers 2068970 (for **2 a**), 2068971 (for **2 d**) and 2068972 (for **3 f**) contain the supplementary crystallographic data for this paper. These data are provided free of charge by the joint Cambridge Crystallographic Data Centre and Fachinformationszentrum Karlsruhe Access Structures service www.ccdc.cam.ac.uk/structures.

## Conflict of interest

The authors declare no conflict of interest.

## Supporting information

As a service to our authors and readers, this journal provides supporting information supplied by the authors. Such materials are peer reviewed and may be re‐organized for online delivery, but are not copy‐edited or typeset. Technical support issues arising from supporting information (other than missing files) should be addressed to the authors.

Supporting Information
