# Supplementary material for: Subphthalocyaninato Boron(III) Hydride: Synthesis, Structure and Reactivity
Source: Chemistry. 2021 Jul 7;27(47):12058–62. doi: 10.1002/chem.202101991 (PMC8456786; doi:10.1002/chem.202101991)
Supplement: Supplementary file 1 — Supporting Information [file CHEM-27-12058-s001.pdf]

Chemistry–A European Journal

Supporting Information

Subphthalocyaninato Boron(III) Hydride: Synthesis, Structure and Reactivity

Lara Tejerina[+], Jorge Labella[+], Lara Martínez-Fernández, Inés Corral, M. Victoria Martínez-Díaz,* and Tomás Torres*

Table of content

1. Experimental Section 2
1.1. General methods 2
1.2. Experimental procedures and characterization of SubPc-hydrides **2** 4
1.2.1. Boron (III) Subphthalocyanine Hydrides 4
1.2.2. General methods for the hydroboration of aldehydes with SubPc **2a** 13
1.2.3. Experiment of decomposition of SubPc **3f** by TMSOTf. 18
1.3. NMR and mass spectra of Subphthalocyanines **3a-3h** 22
1.4. Reaction Mechanism considerations 38
1.4.1. Borenium catalysis 38
1.4.2. TMSOTf Catalysis: 38
2. Computational study 39
2.1. Aldehyde coordination mechanism 40
2.2. Hydride transfer mechanisms 40
2.2.1. Reactants 40
2.2.2. Transition states 41

1. Experimental Section

1.1. General methods

Chemical reagents were purchased from Aldrich or TCI Europe and were used without further purification. *Synthetic grade* solvents were used for chemical reactions and column chromatography purifications and *anhydrous grade* for reactions under dry conditions. Starting SubPcs **1a**, **1b**, **1c** and **1d** were prepared by previously described methods.[1]

The monitoring of the reactions has been carried out by thin layer chromatography (TLC), employing aluminium sheets coated with silica gel type 60 F_{254} (0.2 mm thick, Merck). The analysis of the TLCs was carried out with an UV lamp of 254 and 365 nm. Purification and separation of the synthesized products was performed by normal-phase column chromatography, using silica gel (230-400 mesh, 0.040-0.063 mm, Merck). Eluents along with the relative ratio in the case of solvent mixtures are indicated for each case.

Melting points were measured in open-end capillary tubes by using a Büchi B-540 apparatus and are uncorrected.

Nuclear magnetic resonance spectra (^{1}H-, ^{13}C-, ^{19}F- and ^{11}B-NMR) were recorded on a Bruker AV-300 or a Bruker DRX-500 spectrometer either in the Organic Chemistry Department or in the

[1] a) C. G. Claessens, D. González-Rodríguez, B. del Rey, T. Torres, G. Mark, H-P. Schuchmann, C. von Sonntag, J. G. MacDonald, R. S. Nohr, *Eur. J. Org. Chem.* **2003**, 2547. b) C. G. Claessens, T. Torres, *Eur. J. Org. Chem.* **2000**, 1603.

Interdepartmental Investigation Service of UAM. Deuterated solvent employed in each case is indicated in brackets, and its residual peak was used to calibrate the spectra using literature reference δ ppm values.[2]

Mass spectra (MS) and high-resolution mass spectra (HRMS) were recorded in the Interdepartmental Investigation Service of UAM, employing Atmospheric Pressure Chemical ionization (APCI) using a Bruker-MAXIS II or Matrix-Assisted Laser Desorption/Ionization Time-Of-Flight (MALDI-TOF), using Bruker-Ultraflex-III spectrometer, with a Nd:YAG laser operating at 355 nm. The matrixes and internal references employed are indicated for each spectrum.

Ultraviolet and visible (UV-Vis) spectra were recorded using solvents in the *spectroscopic grade* in the Organic Chemistry Department of UAM employing a JASCO-V660 spectrophotometer. The logarithm of the molar extinction coefficient (ε) is indicated in brackets for each maximum. Likewise, fluorescence measurements were carried out with a JASCO-V8600 spectrofluorometer. Fluorescence quantum yields (ϕ_F) of SubFlcs were determined in toluene and calculated by using the following equation:[3]

$$\phi_F^S = \phi_F^R \left(\frac{Grad_S}{Grad_R}\right)\left(\frac{\eta_S}{\eta_R}\right)^2$$

Scripts R and S indicate reference and sample, respectively. *Grad* is the gradient from the plot of the integrated fluorescence intensity (at exc. λ= 520 nm) *versus* the absorption (at the same wavelength), and η is the refractive index of the solvent. Chloro-dodecafluoroSubPc (Cl-F_{12}SubPc) in benzonitrile (ϕ_F = 0.58) was used as reference.[4]

Infrared spectra were recorded in the Interdepartmental Investigation Service of UAM on a Bruker IFS 66v vacuum FT-IR spectrometer.

Electrochemical measurements were performed in the Organic Chemistry Department of UAM on an Autolab PGStat 30 equipment using a three-electrode configuration system. The measurements were carried out in argon saturated THF solutions containing 0.1 M tetrabutylammonium hexafluorophosphate ($TBAPF_6$). A platinum electrode (3 mm diameter) was used as the working electrode, and a platinum wire and a Ag/$AgNO_3$ (0.01 M in acetonitrile) electrode were employed as the auxiliary and the reference electrode, respectively. Fc was used as an external reference and all the potentials were given relative to the Fc/Fc^+ couple.

Single-crystal X-ray diffraction data collection for structure determinations were collected in the Interdepartmental Investigation Service of UAM at Bruker KAPPA APEX II CCD area-detector X-ray diffractometer operating with graphite-monochromated and Mo Kalpha radiation (λ = 0.71073 Å). The data are absorption corrected with the program SADABS. Intensities are calculated with the SAINT software, which also incorporates polarization and Lorentz effect corrections. The structures were solved and refined using the Bruker SHELXTL Software Package.

---

[2] G. R. Fulmer, A. J. M. Miller, N. H. Sherden, H. E. Gottlieb, A. Nudelman, B. M. Stoltz, J. E. Bercaw, K. I. Goldberg, *Organometallics* **2010**, *29*, 2176.

[3] J. R. Lakowicz, in *Principles of fluorescence spectroscopy*, 3rd ed. Springer, New York, **2006**.

[4] C. Romero-Nieto, J. Guilleme, C. Villegas, J. L. Delgado, D. González-Rodríguez, N. Martin, T. Torres, D. M. Guldi, *J. Mater. Chem.* **2011**, *21*, 15914.

1.2. Experimental procedures and characterization of SubPc-hydrides 2

1.2.1. Boron (III) Subphthalocyanine Hydrides

<u>Boron (III) Subphthalocyanine hydride **2a**</u>

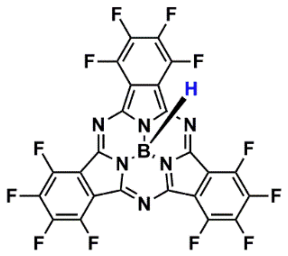

SubPc **1a** (97 mg, 0.15 mmol) was dissolved in freshly distilled toluene (3 ml) under an argon atmosphere. The solution was degassed by freeze-pump-thaw method. After that, the reaction mixture was cooled down at -55 °C, and then DIBAL-H was added dropwise (1 M in THF, 0.75 ml). The reaction mixture was stirred for 3 min and filtered through a short silica plug. The solvent was removed under vacuum to yield SubPc **2a** as a pink solid (**45 mg, 49%**)

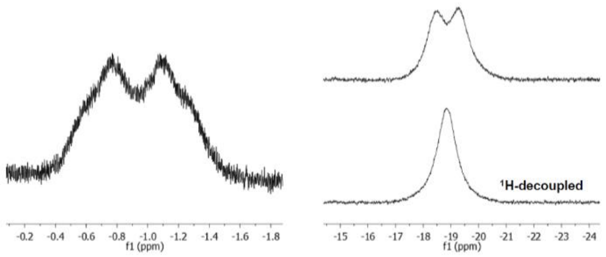

Figure S1. ^{1}H-NMR (500 MHz, left) and ^{11}B-NMR (160 MHz, right) spectra of SubPc **2a** in $CDCl_3$ at 55 °C.

Mp > 250 °C. **^{1}H-NMR** (500 MHz, CDCl3, 328 K): δ (ppm) = -0.92 (br q, J_{BH} = 155 Hz, 1H, BH). **^{11}B-NMR** (160 MHz, CDCl3, 328 K): δ (ppm) = -18.85 (d). **^{13}C-NMR** (125 MHz, $CDCl_3$, 298 K): δ (ppm) = 148.2, 144.0 – 143.3 (m, $C_{\alpha}F$), 141.8 – 141.2 (m, $C_{\beta}F$), 114.9 – 114.8 (m). **^{19}F-NMR** (470 MHz, $CDCl_3$, 298 K): δ (ppm) = -137.4 (m), -148.1 (m). **UV-Vis (THF):** λmax (nm) (log ε) = 573 (4.89), 535 (sh), 518 (sh), 307 (4.56). **FT-IR (KBr):** *ν* (cm−1) = 2932, 2858, 2479 (*ν* BH), 2434 (*ν* BH), 1646, 1533, 1487, 1420, 1340, 1262, 1224, 1165, 1126, 1079, 1057, 1018, 961, 827, 770, 742, 699, 640, 579. **MS (MALDI-TOF, DCTB):** m/z = 612.1 [M]+, 611.1 $[M-H]^+$. **HRMS (MALDI-TOF, DCTB+PPG450+790)** ($C_{24}HBF_{12}N_6$): $[M]^+$: Calculated: 612.0163; Found: 612.0165

XRD: Selected crystallographic data for SubPc **2a**. The single crystal analyzed was obtained by slow evaporation of **2a** in $CHCl_3$. CCDC: 2068970

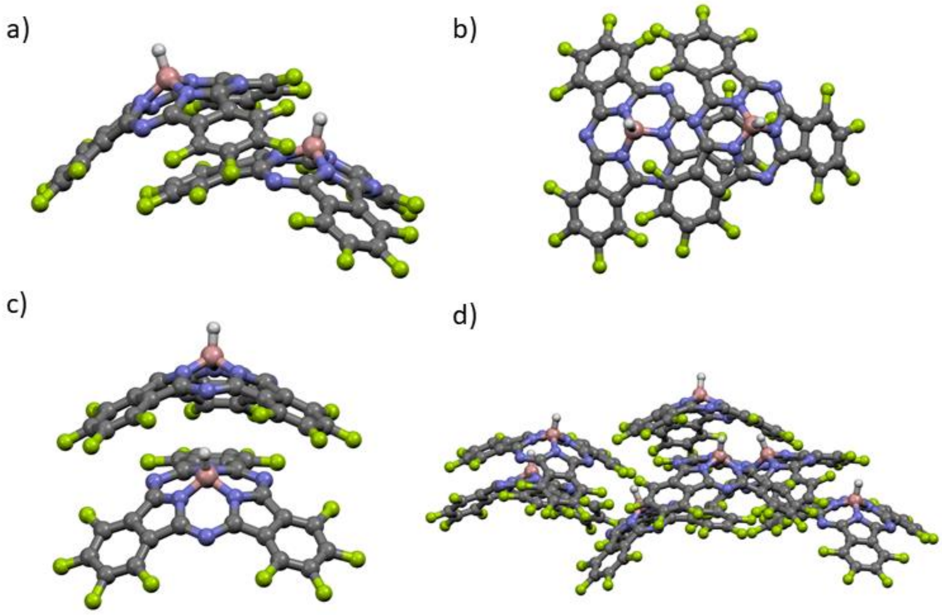

Figure S2. a) front, b) top and c) side view of head-to-tail dimers of **2a.** d) packing of **2a**

Chemical formula	**$C_{24.33}H_{1.33}BClF_{12}N_6$**	
Formula weight	651.91 g/mol	
Temperature	200(2) K	
Wavelength	0.71073 Å	
Crystal size	0.010 x 0.081 x 0.231 mm	
Crystal habit	purple plate	
Crystal system	trigonal	
Space group	R 3 c	
Unit cell dimensions	a = 34.863(5) Å	α = 90°
	b = 34.863(5) Å	β = 90°
	c = 9.8013(17) Å	γ = 120°
Volume	10317.(4) $Å^3$	
Z	18	
Density (calculated)	1.889 g/cm^3	
Absorption coefficient	0.297 mm^{-1}	
F(000)	5748	
Goodness-of-fit on F^2	1.010	
Final R indices	I>2σ(I) R1 = 0.0571, wR2 = 0.1283	

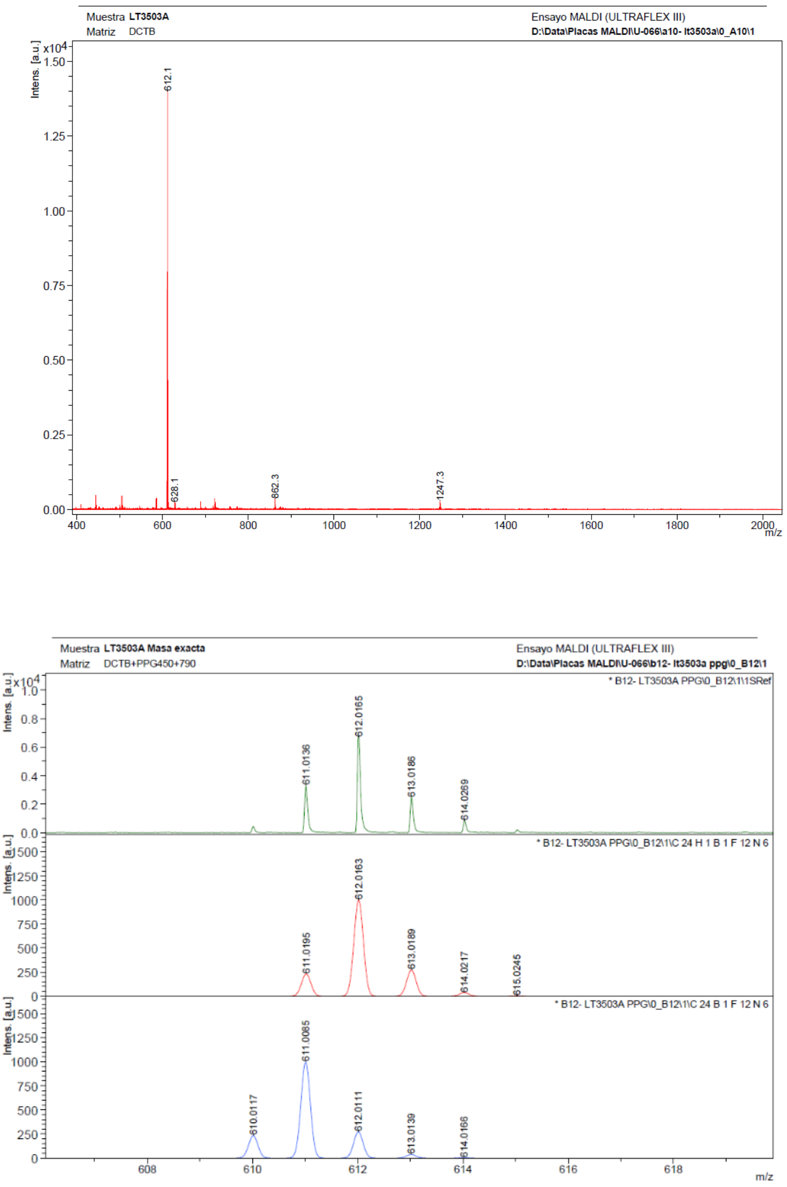

Figure S3. High-resolution mass spectrum of SubPc **2a**

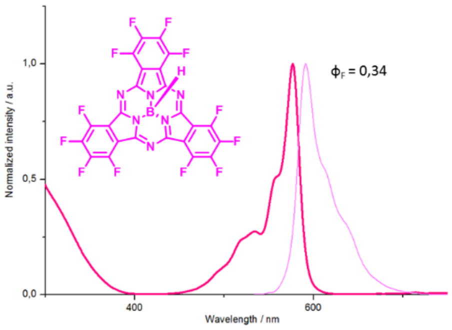

Figure S4. UV-vis (solid line) and fluorescence (thin line) spectra in toluene of SubPc **2a** (exc. λ = 520 nm). Fluorescence quantum yields (φ_F) are indicated.

Table S1. Electrochemical data of SubPc **2a** determined by SWV.

E_{Ox1} [V] *vs* Fc/Fc^{+}	E_{Red1} [V] *vs* Fc/Fc^{+}	E_{0-0} [nm]	E_{0-0} [eV][a]	E_{HOMO} [eV][b]	E_{LUMO} [eV]
0.89[e]	-1.04[d]	579	2.1	-6.0[b] (-6.2)[c]	-4.1[b]

[a] The zero-zero excitation energy (E_{0-0}) is converted to eV according to the relation E_{0-0} (eV) = 1240/E_{0-0} (nm). [b] Calculated applying Eq 1. [c] Calculated applying Eq 2. [d] Reversible. [e] Irreversible.

$$E_{HOMO/LUMO}(eV) = -5.1 - E_{1Ox/Red}(vs\, Fc/Fc^{+}) \quad \text{(Eq 1)}$$

$$E_{HOMO}(eV) = E_{LUMO} - E_{0-0}(eV) \quad \text{(Eq 2)}$$

Boron (III) Subphthalocyanine hydride **2b**

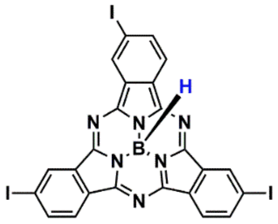

SubPc **1b** (50 mg, 0.062 mmol) was dissolved in freshly distilled toluene (1.5 ml) under an argon atmosphere. The solution was degassed by freeze-pump-thaw method. After that, the reaction mixture was cooled down at -55 °C, and then DIBAL-H was added dropwise (1 M in THF, 0.62 ml). The reaction mixture was stirred for 3 min and filtered through a short silica plug. The solvent was removed under vacuum and washed with hexane and methanol to yield SubPc **2b** as a pink solid. (**21 mg, 44%**)

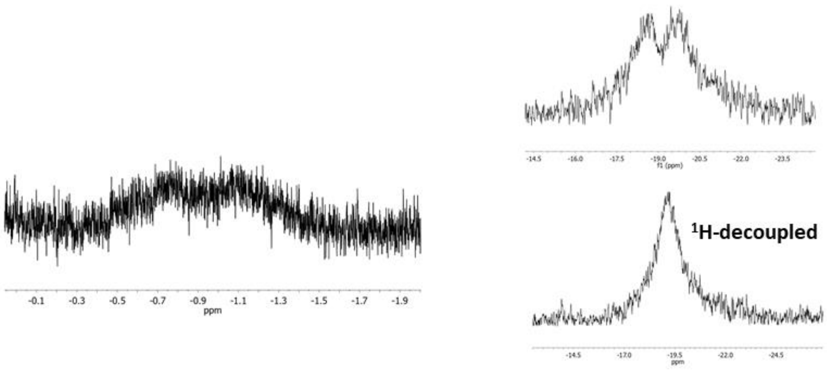

Figure S5. ^{1}H-NMR (500 MHz, left) and ^{11}B-NMR (160 MHz, right) spectra of SubPc **2b** in CDCl3 at 55ºC

Mp > 250 °C.**^{1}H-NMR** (500 MHz, CDCl3, 328 K): δ (ppm) = 9.18 (br s, 3H), 8.52 (m, 3H), 8.19 (m, 3H) -0.94 (br q, J_{BH} = 155 Hz, 1H, BH). **^{13}C-NMR**: the low solubility of this compound precludes its characterization by ^{13}C-NMR. **^{11}B-NMR** (160 MHz, CDCl3, 328 K): δ (ppm) = -19.21 (d). **UV-Vis (Toluene):** λmax (nm) (log ε) = 574 (4.8), 517 (sh), 321 (4.2).**FT-IR (KBr)**: ν (cm−1) = 3115,2935, 2898, 2423 (ν BH), 1604, 1586, 1444, 1184, 1189, 1148, 1101, 1058, 990,

820, 781, 686. **MS (MALDI-TOF, DCTB):** m/z = 773.9 $[M]^+$, 772.9 $[M-H]^+$. **HRMS (MALDI-TOF, DCTB+PPG450+790)** ($C_{24}H_{10}BI_3N_6$): $[M]^+$: Calculated: 773.8193; Found: 773.8189

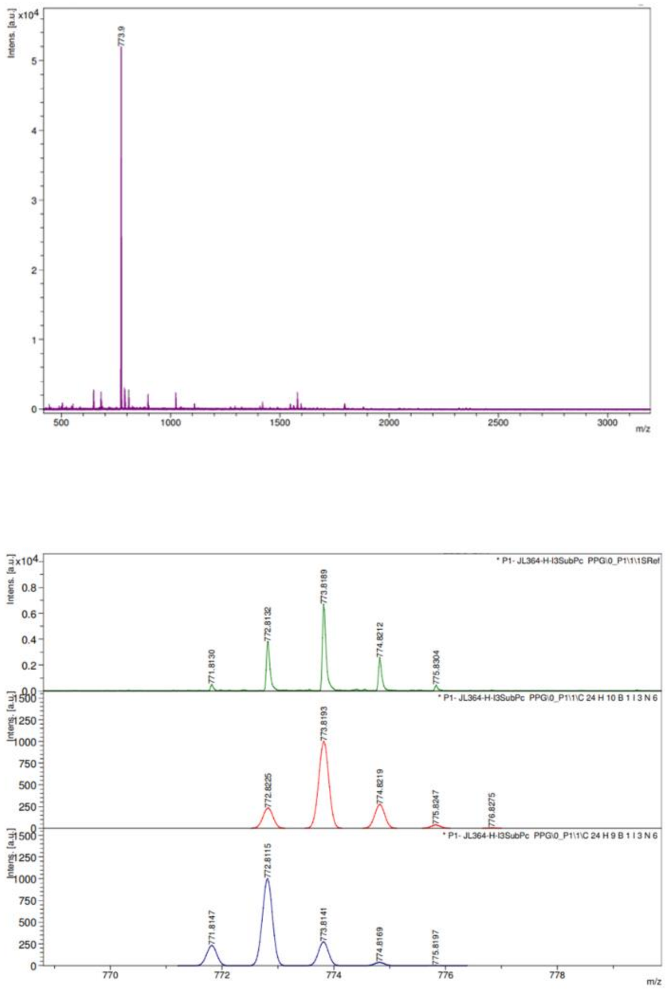

Figure S6. High-resolution mass spectrum of SubPc **2a**

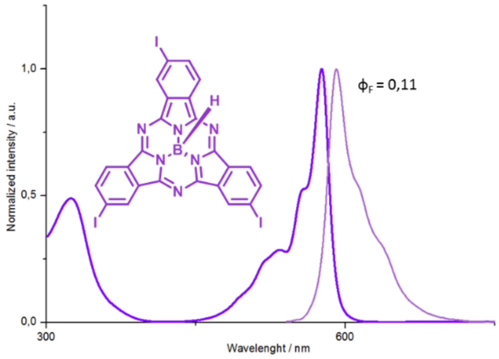

Figure S7. UV-vis (solid line) and fluorescence (thin line) spectra in toluene of SubPc **2b** (exc. λ = 520 nm). Fluorescence quantum yields (φ_F) are indicated.

Boron (III) Subphthalocyanine hydride **2c**

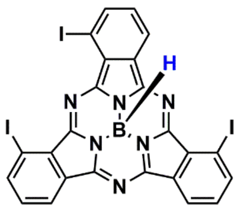

SubPc **1c** (50 mg, 0.062 mmol) was dissolved in freshly distilled toluene (1.5 ml) under an argon atmosphere. The solution was degassed by freeze-pump-thaw method. After that, the reaction mixture was cooled down at -55 °C, and then DIBAL-H was added dropwise (1 M in THF, 1.9 ml, 30 eq). The reaction mixture was stirred for 3 min and filtered through a short silica plug. The solvent was removed under vacuum and washed with hexane and methanol to yield SubPc **2d** as a pink solid. (**19 mg, 40%**)

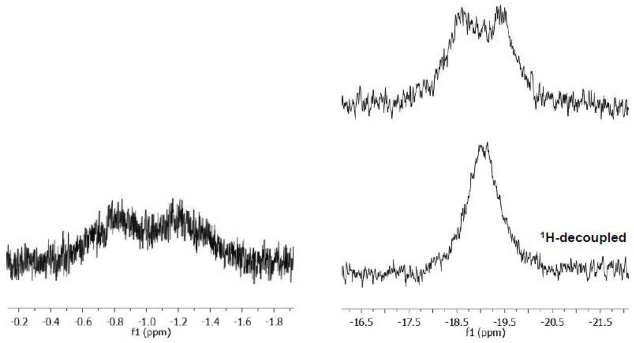

Figure S8. ^{1}H-NMR (500 MHz, left) and ^{11}B-NMR (160 MHz, right) spectra of SubPc **2c** in CDCl3 at 55 °C.

Mp > 250 °C. **^{1}H-NMR** (500 MHz, CDCl3, 328 K): δ (ppm) = 8.83 – 8.77 (m, 3H), 8.32 – 8.28 (m, 3H), 7.54 – 7.47 (m, 3H) -1.02 (br q, J_{BH} = 155 Hz, 1H, BH). **^{13}C-NMR**: the low solubility of this compound precludes its characterization by ^{13}C-NMR. **^{11}B-NMR** (160 MHz, CDCl3, 328 K): δ (ppm) = - 19.14 (d). **UV-Vis (THF):** λmax (nm) (log ε) = 575 (4.7), 521 (sh), 301 (4.1). **FT-IR (KBr):** *ν* (cm^{-1}) = 2958, 2923, 2854, 2484 (*ν* BH), 1725, 1650, 1612, 1604, 1544, 1453, 1402, 1366, 1313, 1244, 1192, 1132, 1111, 1060, 1043, 907, 782, 750, 722, 683, 613, 572 **MS (MALDI-TOF, DCTB):** m/z = 773.9 $[M]^{+}$, 772.9 $[M-H]^{+}$. **HRMS (MALDI-TOF, DCTB+PPG450+790)** ($C_{24}H_{10}BI_3N_6$): $[M]^{+}$: Calculated: 773.8193; Found: 73.8183.

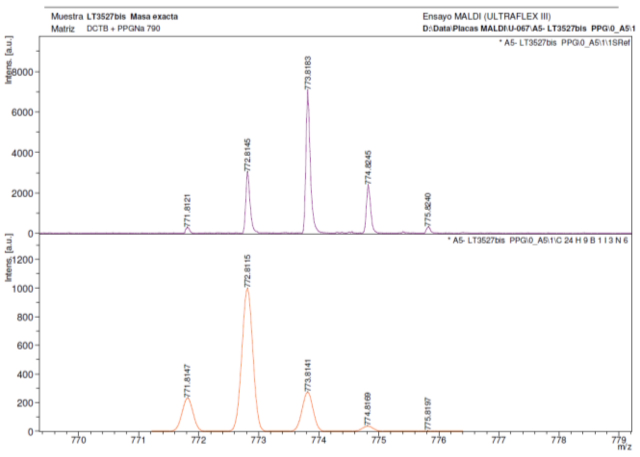

Figure S9. High-resolution mass spectrum of SubPc **2c**

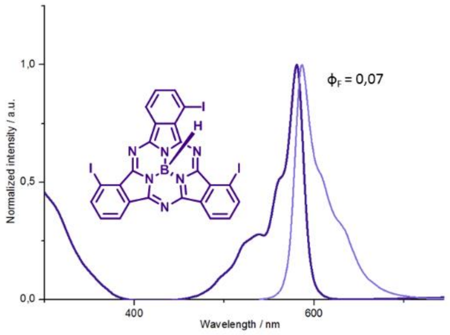

Figure S10. UV-vis (solid line) and fluorescence (thin line) spectra in toluene of SubPc **2c** (exc. λ = 520 nm). Fluorescence quantum yields (φ_F) are indicated.

<u>Boron (III) Subphthalocyanine hydride **2d**</u>

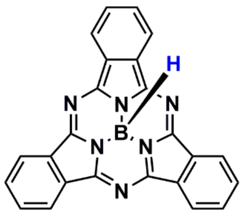

SubPc **1d** (100 mg, 0.232 mmol) was dissolved in freshly distilled toluene (3ml) under an argon atmosphere. The solution was degassed by freeze-pump-thaw method. After that, the reaction was kept at room temperature and then DIBAL-H was added dropwise (1 M in THF, 4.7 ml). The reaction mixture was stirred for 40 min and filtered through a short silica plug. The solvent was removed under vacuum to yield SubPc **2d** as a pink solid. (**1.9 mg, 2%**)

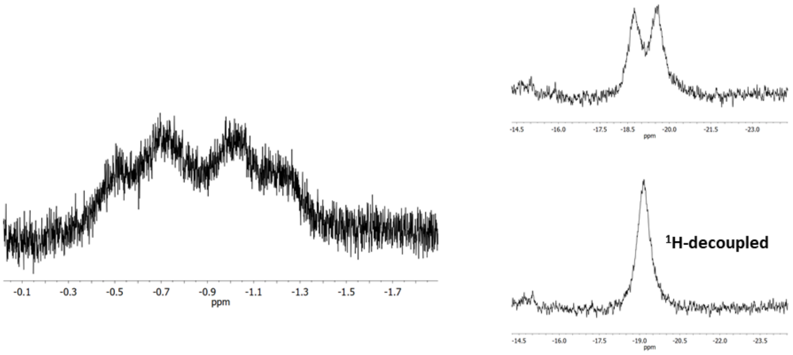

Figure S11. ^{1}H-NMR (500 MHz, left) and ^{11}B-NMR (160 MHz, right) spectra of SubPc **2d** in CDCl3 at 55 °C.

Mp > 250 °C.**^{1}H-NMR** (500 MHz, CDCl3, 328 K): δ (ppm) = 8.86 (m,6H), 7.88 (m,6H), -0.90 (br q, J_{BH} = 155 Hz, 1H, BH). **^{13}C-NMR**: the low solubility of this compound precludes its characterization by ^{13}C-NMR. **^{11}B-NMR** (160 MHz, CDCl3, 328 K): δ (ppm) = - 19.12(d). **UV-Vis (Toluene):** λmax (nm) (log ε) = 564 (4.7), 523 (sh), 302 (4.2) **MS (MALDI-TOF, DCTB):** m/z = 396.2 $[M]^{+}$, 395.2 $[M-H]^{+}$. **HRMS (MALDI-TOF, DCTB+PPG450+790)** ($C_{24}H_{13}BN_6$): $[M]^{+}$: Calculated: 396.1294; Found: 396.1311

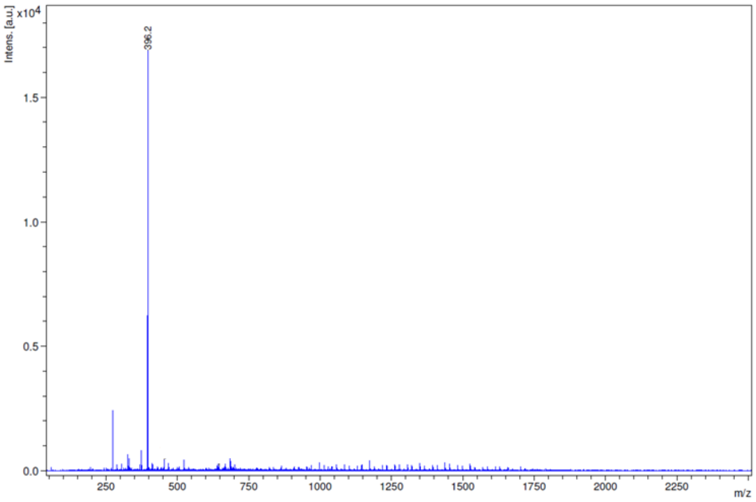

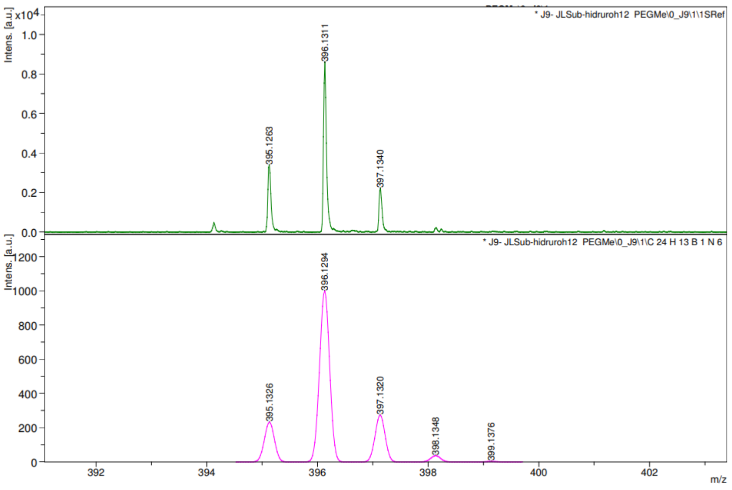

Figure S12. High-resolution mass spectrum (MALDI-TOF, DCTB) of SubPc **2d**.

XRD: Selected crystallographic data and some refining details are summarized in the following table. The single crystal analyzed was obtained by slow evaporation of **2d** in CH_2Cl_2. CCDC: 2068971

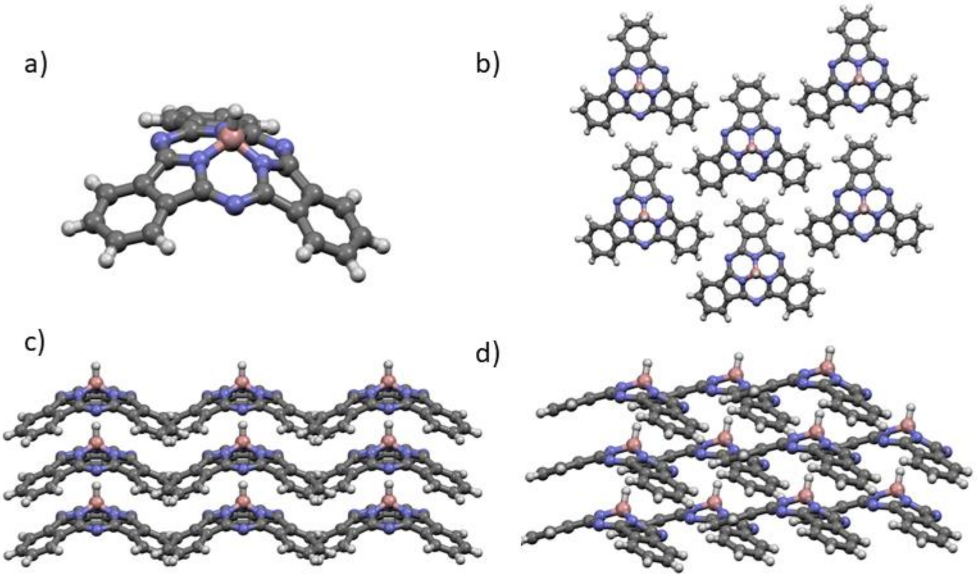

Figure S13. a) X-Ray structure of SubPc **2d**. b) top, c) front and c) side view of **2d** packing

Chemical formula	$\mathbf{C_{24}H_{13}BN_6}$	
Formula weight	396.21 g/mol	
Temperature	250(2) K	
Wavelength	0.71073 Å	
Crystal size	0.035 x 0.041 x 0.254 mm	
Crystal habit	purple needle	
Crystal system	trigonal	
Space group	R 3 m	
Unit cell dimensions	a = 19.4398(5) Å	α = 90°
	b = 19.4398(5) Å	β = 90°

	c = 4.07370(10) Å	γ = 120°
Volume	1333.22(8) $Å^3$	
Z	3	
Density (calculated)	1.480 g/cm^3	
Goodness-of-fit on F2	1.188	
Final R indices	563 data; I>2σ(I)	R1 = 0.0294, wR2 = 0.0754
	all data	R1 = 0.0311, wR2 = 0.0762

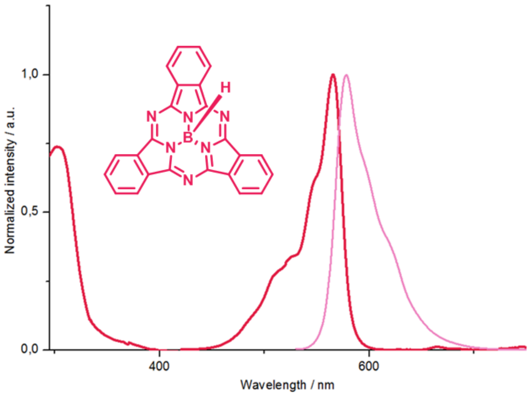

Figure S14. UV-vis (solid line) and fluorescence (thin line) spectra in toluene of SubPc **2d** (exc. λ = 520 nm).

1.2.2. General methods for the hydroboration of aldehydes with SubPc 2a

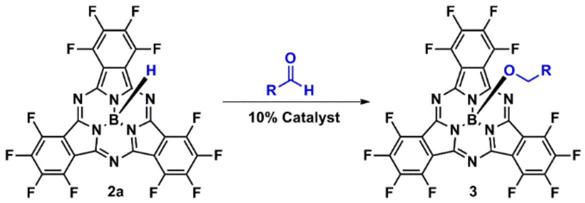

Method A: Catalysis with TMSOTf

SubPc **2a** (10 mg, 16 µmol), the appropriate aldehyde (16 µmol) and trimethylsilyl trifluoromethanesulfonate (0.3 µL, 10 mol%) were dissolved in freshly distilled toluene (1 ml) under argon atmosphere. The reaction mixture was stirred at 50 °C until the starting SubPc **2a** is consumed.[5] The solvent was then eliminated under reduced pressure and the residue purified by column chromatography on silica gel (the eluent and the time of reaction are specified in each case).

Method B: Catalysis with $SubPcB^+$

SubPc **2a** (11 mg, 17.6 µmol), the appropriate aldehyde (16 µmol) and trityl tetrakis (pentafluorophenyl)borate (1.5 mg, 1.6 µmol), were dissolved in freshly distilled toluene (1 ml) under argon atmosphere. The reaction mixture was stirred at 55 °C until the starting SubPc **2a** is

[5] The disappearance of SubPc **2a** can be easily monitored by TLC. Under not rigorously anhydrous conditions or long reaction times, by-products such as hydroxy-SubPc or µ-oxo dimer are detected.

consumed.[5] The solvent was then eliminated under reduced pressure and the residue purified by column chromatography on silica gel (the eluent and the time of reaction are specified in each case).

Boron (III) Subphthalocyanine **3a**

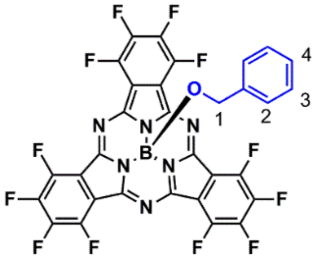

Method A: TMSOTf.
Reaction time: 25 min**.** Eluent: Toluene. Yield: **10.9 mg, 93%**
Method B: SubPcB$^+$
Reaction time: 80 min Eluent: Toluene. Yield: **10.8 mg, 92%**

Mp > 250 ºC**.** **^{1}H-NMR** (300 MHz, $CDCl_3$, 298 K): δ (ppm) = 6.99 (m, 3H, H3-4), 6.39 (m, 2H, H2), 2.69 (s, 2H, H1). **^{13}C-NMR** (76 MHz, $CDCl_3$, 298 K): δ (ppm)= 148.49, 144.46, 140.94, 138.19, 128.08, 127.19, 125.92, 115.11, 62.25. **MS (MALDI-TOF, DCTB)** [M]+**:** m/z = 718.0. **APCI-HRMS** ($C_{31}H_8BF_{12}N_6O$) [M+H]$^+$: Calculated: 719.0661; Found: 719.0670. **UV-Vis (Toluene):** λmax (nm) (log ε) = 571 (4.5), 535 (sh), 518 (sh), 307 (4.56).

Boron (III) Subphthalocyanine **3b**

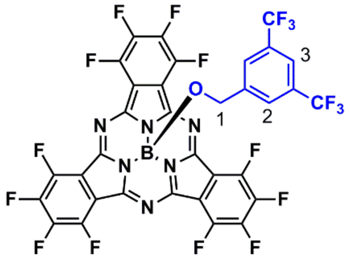

Method A: TMSOTf.
Reaction time: 120 min. Eluent: Toluene/ Heptane (3:1) Yield: **10.2 mg, 73%**

Mp > 250 ºC**.** **^{1}H-NMR** (300 MHz, $CDCl_3$, 298 K): δ (ppm) = 7.51 (s, 1H, H3), 6.87 (s, 2H, H2), 2.73 (s, 2H, H1). **^{13}C-NMR** (76 MHz, $CDCl_3$, 298 K): δ (ppm)= 148.65, 144.48, 141.07, 140.89, 131.44 (q, $^2J_{C-F}$= 33.6 Hz), 125.70, 123.12 (q, $^1J_{C-F}$= 274.6 Hz), 121.11, 115.06, 60.59. **^{19}F-NMR** (471 MHz, $CDCl_3$, 298 K): δ (ppm)= -63.15 (s), -136.69 (m), -147.31 (m). **MS (MALDI-TOF, DCTB):** m/z = 854.2 [M]+. **APCI-HRMS** ($C_{33}H_6BF_{18}N_6O$) [M+H]$^+$: Calculated: 855.0409; Found: 855.0415**. UV-Vis (Toluene):** λmax (nm) (log ε) = 572 (4.5), 554 (sh), 525 (sh), 303 (4.3).

Boron (III) Subphthalocyanine **3c**

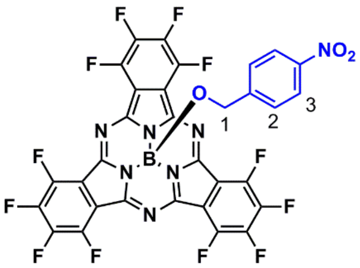

Method A: TMSOTf.

Reaction time: 240 min**.** Eluent: Toluene. Yield: **9.9 mg, 81%**

Mp > 250 ºC**.** **^{1}H-NMR** (500 MHz, $CDCl_3$, 298 K): δ (ppm) = 7.84 (m, 2H, H3), 6.55 (d, J_o = 8.7 Hz, 2H, H2), 2.71 (s, 2H, H1). **^{13}C-NMR** (126 MHz, $CDCl_3$, 298 K): δ (ppm)= 148.71, 147.02, 145.64, 143.75, 141.50, 125.99, 123.31, 115.11, 60.69. **MS (MALDI-TOF, DCTB):** m/z = 763.1 [M]+. **APCI-HRMS** ($C_{31}H_7BF_{12}N_7O_3$) $[M+H]^+$: Calculated: 764.0512; Found: 764.0524**. UV-Vis (Toluene):** λmax (nm) (log ε) = 571 (4.6), 556 (sh), 528 (sh), 301 (4.5).

Boron (III) Subphthalocyanine **3d**

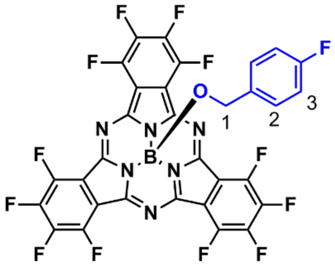

Method A: TMSOTf**.**

Reaction time: 120 min**.** Eluent: Toluene. Yield: **8.9 mg, 74%**

Mp > 250 ºC**.** **^{1}H-NMR** (500 MHz, $CDCl_3$, 298 K): δ (ppm) = 6.69 (t, J_o = 8.7 Hz, 2H, H3), 6.37 (dd, $J_{oH\text{-}H}$ = 8.7 Hz, $J_{mF\text{-}H}$ = 6Hz, 2H, H2), 2.63 (s, 2H, H1). **^{13}C-NMR** (126 MHz, $CDCl_3$, 298 K): δ (ppm)= 161.9 (d, $^1J_{C\text{-}F}$= 245.7 Hz), 148.56, 143.48, 141.41, 133.90 (d, $^4J_{C\text{-}F}$= 3.8 Hz), 127.56 (d, $^3J_{C\text{-}F}$= 8 Hz), 115.05, 114.94 (d, $^2J_{C\text{-}F}$= 22.7 Hz), 61.41. **^{19}F-NMR** (471 MHz, $CDCl_3$, 298 K): δ (ppm)= -115.09 (s), -137.00 (m), -147.57 (m). **MS (MALDI-TOF, DCTB):** m/z = 736.1 [M]+. **APCI-HRMS** ($C_{31}H_7BF_{13}N_6O$) $[M+H]^+$:Calculated: 737.0567; Found: 764.0524. **UV-Vis (Toluene):** λmax (nm) (log ε) = 571 (4.6), 530 (sh), 303 (4.5).

Boron (III) Subphthalocyanine **3e(Carbono)**

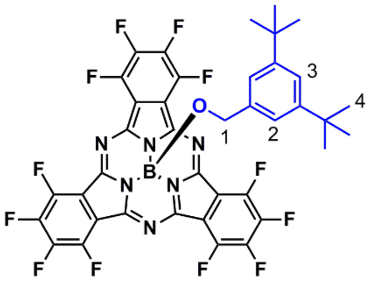

Method A: TMSOTf.

Reaction time: 30 min. Eluent: Toluene/ Heptane (3:1). Yield: **13.6 mg, 89%**

^{1}H-NMR (300 MHz, $CDCl_3$, 298 K): δ (ppm) = 7.10 (s, 1H, H3), 6.20 (s, 2H, H2), 2.74 (s, 2H, H1), 1.18 (s, 18H, H4). **^{13}C-NMR** (76 MHz, $CDCl_3$, 298 K): δ (ppm)= 150.7, 148.4, 143.6, 141.5, 137.2, 121.6, 120.2, 115.1, 63.3, 34.7, 31.4. **MS (MALDI-TOF, DCTB):** m/z = 830.2 [M]+. **HRMS (MALDI-TOF, DCTB+PPG790)** ($C_{39}H_{23}BF_{12}N_6O$)[M]+: Calculated:830.1836; Found: 830.1853. **UV-Vis (Toluene):** λmax (nm) (log ε) = 571 (4.5), 535 (sh), 518 (sh), 305 (4.4).

<u>Boron (III) Subphthalocyanine **3f**</u>

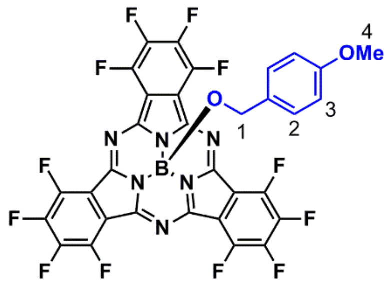

<u>Method B: SubPcB^{+}</u>

Reaction time: 60 min. Eluent: Toluene. Yield: **8.2 mg, 67%**

^{1}H-NMR (300 MHz, $CDCl_3$, 298 K): δ (ppm) = 6.53 (d, J_o = 8.7 Hz, 2H, H2), 6.32 (d, J_o = 8.7 Hz, 2H, H3), 3.66 (s, 3H, H4), 2.65 (s, 2H, H1). **^{13}C-NMR** (76 MHz, $CDCl_3$, 298 K): δ (ppm)= 158.86, 148.42, 144.50, 141.12, 130.24, 127.49, 115.17, 113.47, 62.00, 55.30. **MS (MALDI-TOF, DCTB):** m/z = 748.2 [M]$^{+}$. **APCI-HRMS** ($C_{32}H_{10}BF_{12}N_6O_2$) [M+H]$^{+}$: Calculated: 749.0767; Found: 749.0780. **UV-Vis (Toluene):** λmax (nm) (log ε) = 571 (4.6), 528 (sh), 302 (4.5).

XRD: Selected crystallographic data for SubPc **3f**. The single crystal analyzed was obtained by slow recrystallization of **3f** in CH_2Cl_2/hexane solution. CCDC: 2068972

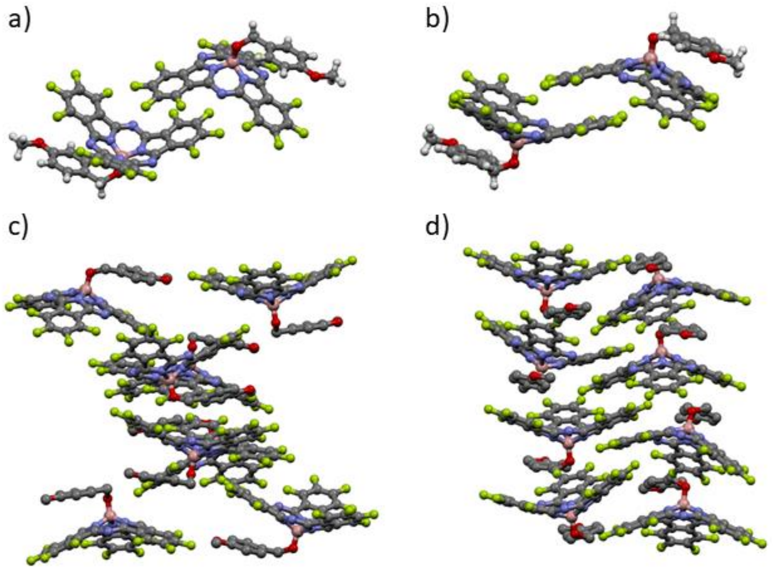

Figure S15. a) X-Ray structure of SubPc and packing of **3f**

**Chemical formula**	**$C_{32}H_9BF_{12}N_6O_2$**

**Formula weight**	748.26 g/mol	
**Temperature**	200(2) K	
**Wavelength**	0.71073 Å	
**Crystal size**	0.010 x 0.172 x 0.198 mm	
**Crystal habit**	clear intense purple plate	
**Crystal system**	monoclinic	
**Space group**	P 1 21/c 1	
**Unit cell dimensions**	a = 13.2839(9) Å	α = 90°
	b = 23.7829(15)Å	β = 109.916(3)°
	c = 19.4204(16) Å	γ = 90°
**Volume**	5768.5(7) $Å^3$	
**Z**	8	
**Density (calculated)**	1.723 g/cm^3	
**Goodness-of-fit on F2**	1.000	
**Final R indices**	4720 data; I>2σ(I)	R1 = 0.0588, wR2 = 0.1165
	all data	R1 = 0.1801, wR2 = 0.1812

Figure S16. X-Ray structure (a) and packing (b) of SubPc **3f**

Boron (III) Subphthalocyanine **3g**

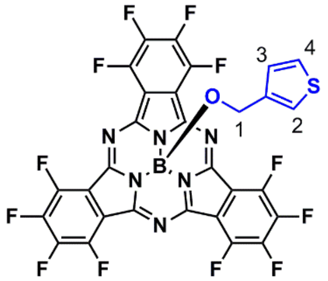

Method B: $SubPcB^+$

Reaction time: 100 min. Eluent: Toluene/ Heptane (3:1). Yield: **9.5 mg, 75%**

^{1}**H-NMR** (300 MHz, $CDCl_3$, 298 K): δ (ppm)= 6.95 (dd, J_o = 5.0 Hz, J_m = 2.9 Hz, 1H, H3), 6.41 (dd, J_m = 2.9 Hz, J_m = 1.1 Hz, 1H, H2), 6.18 (dd, J_o = 5.0 Hz, J_m = 1.1 Hz, 1H, H4) 2.74 (s, 2H, H1). ^{13}**C-NMR** (76 MHz, $CDCl_3$, 298 K): δ (ppm)= 148.45, 144.34, 140.80, 139.50, 126.01, 125.92, 120.99, 115.05, 58.15. **MS (MALDI-TOF, DCTB):** m/z = 724.1 [M]+. **APCI-HRMS** ($C_{29}H_6BF_{12}N_6OS$) $[M+H]^+$; Calculated: 725,0225; Found: 725.0238. **UV-Vis (THF):** λmax (nm) (log ε) = 571 (4.4), 529 (sh), 307 (4.3).

Boron (III) Subphthalocyanine **3h**

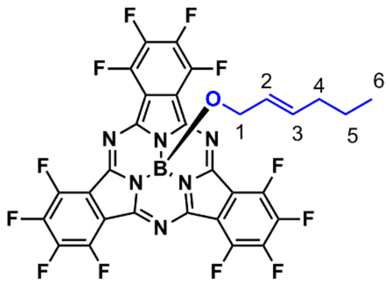

Method A: TMSOTf.

Reaction time: 60 min**.** Eluent: Toluene/ Heptane (3:1)**.** Yield: **8.0 mg, 69%**

^{1}**H-NMR** (300 MHz, $CDCl_3$, 298 K): δ (ppm) = 4.84 (m, 1H, H3), 4.46 (m, 1H, H2), 2.05 (d, J_3 = 3 Hz, 2H, H1), 1.61 (m, 2H, H4), 1.10 (m, J_3 = 3 Hz, 2H, H5), 0.70 (t, J_3 = 3 Hz, 3H, H6). ^{13}**C-NMR** (126 MHz, $CDCl_3$, 298 K): δ (ppm)= 148.60, 143.90, 141.34, 132.36, 126.18, 115.04, 60.90, 34.00, 22.14, 13.66. **MS (MALDI-TOF, DCTB):** m/z = 710.2 [M]+. **APCI-HRMS** ($C_{30}H_{11}BF_{12}N_6O$) $[M+H]^+$: Calculated: 711,0974; Found: 711.0983- **UV-Vis (Toluene):** λmax (nm) (log ε) = 570 (4.3), 529 (sh), 300 (4.2).

1.2.3. Experiment of decomposition of SubPc 3f by TMSOTf.

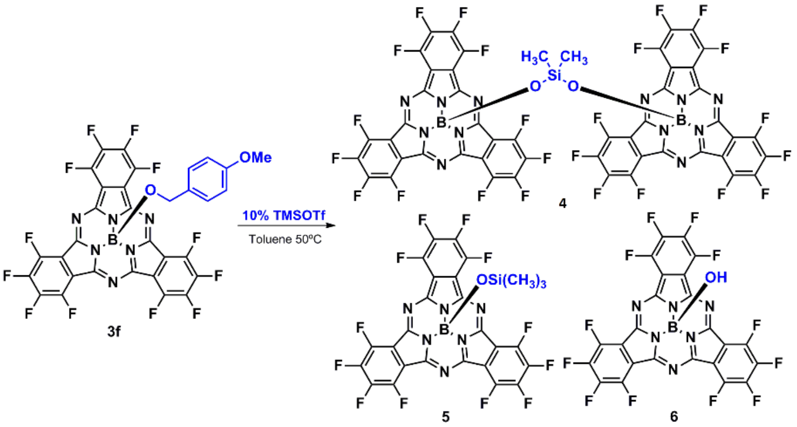

Scheme S1. Decomposition reaction of SubPc **3f** by TMSOTf

SubPc **3f** (10 mg, 13.4 µmol) and trimethylsilyl trifluoromethanesulfonate (10 mol%) were dissolved in dry toluene-d_8 (1 ml) under argon atmosphere. The reaction mixture was stirred at 50 °C and the complete disappearance of **3f** was observed by TLC.

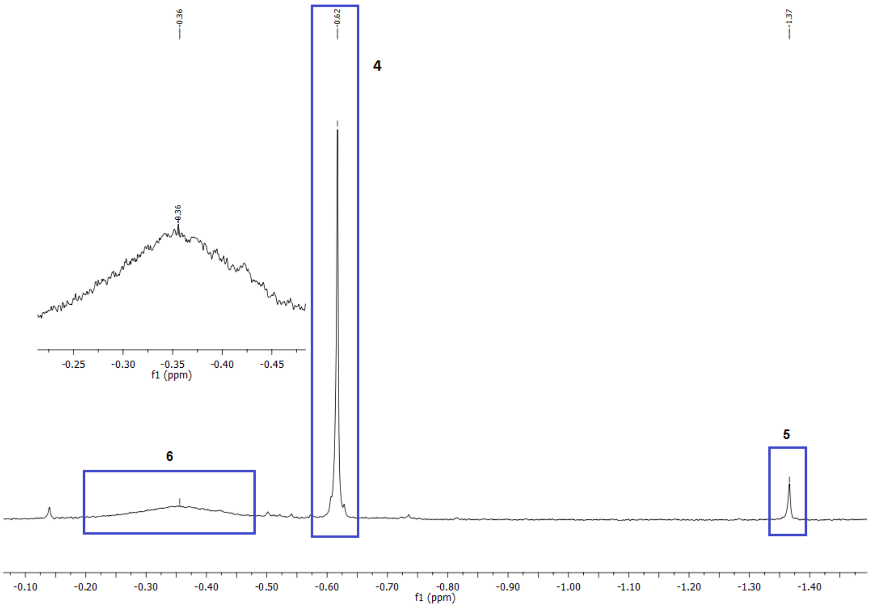

Figure S17. ^{1}H-NMR of reaction mixture in toluene-d_8

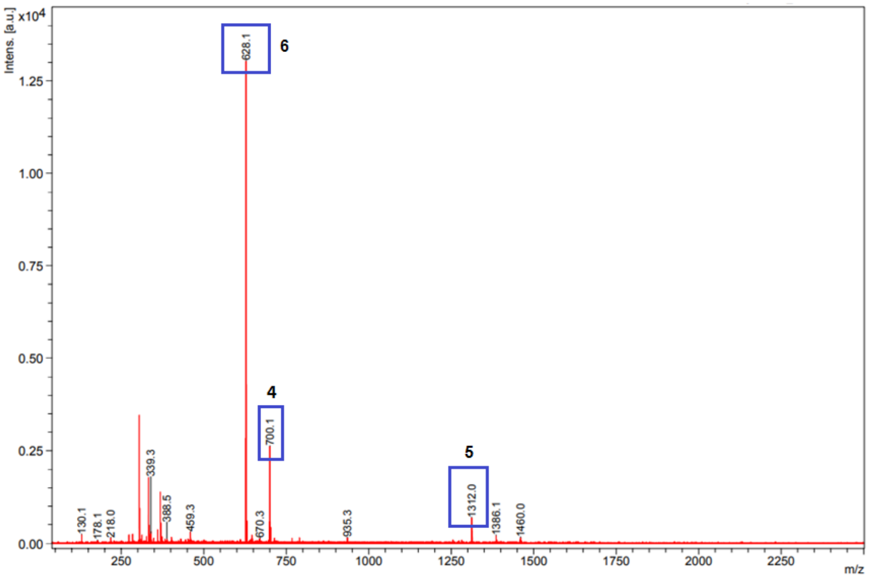

Figure S18. MALDI spectrum of the reaction mixture.

<u>Boron (III) Subphthalocyanine **5**[6]</u>

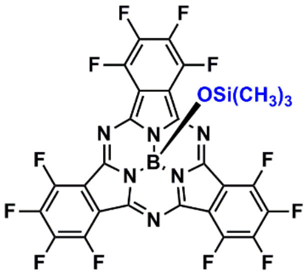

^{1}H-NMR (300 MHz, $CDCl_3$, 298 K): δ (ppm) = -0.79 (s, 9H). **APCI-HRMS** ($C_{27}H_{10}BF_{12}N_6OSi$) $[M+H]^+$: Calculated: 701.0586; Found: 701.0596. **UV-Vis (Toluene):** λmax (nm) (log ε) = 569 (4.7), 527 (sh), 302 (4.5)

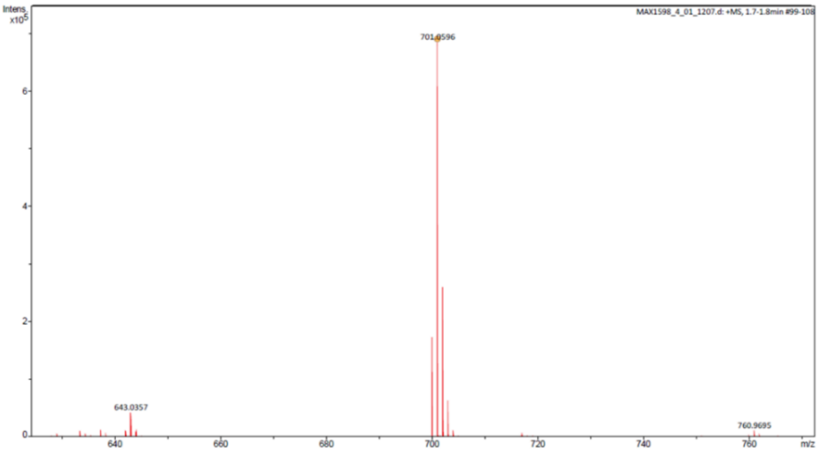

[6] SubPc byproduct in the hydroboration of the aldehydes **g** and **h** under TMSOTf catalysis and purified by column chromatography on silica gel (toluene).

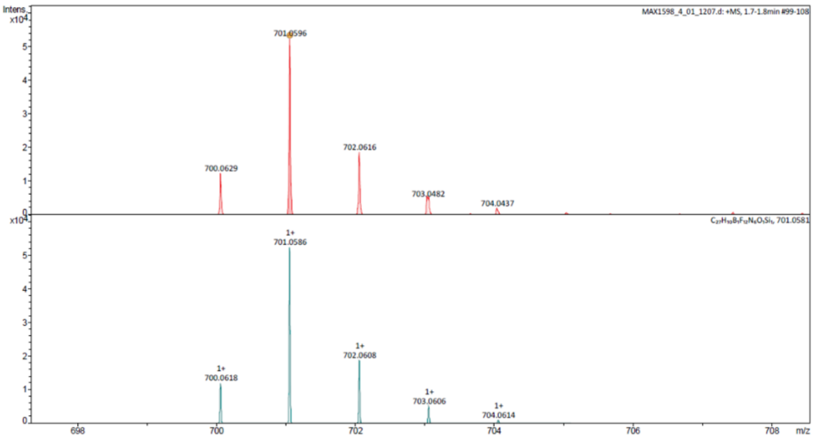

Figure S19. APCI spectra of SubPc **5**

Boron (III) Subphthalocyanine **6**[6]

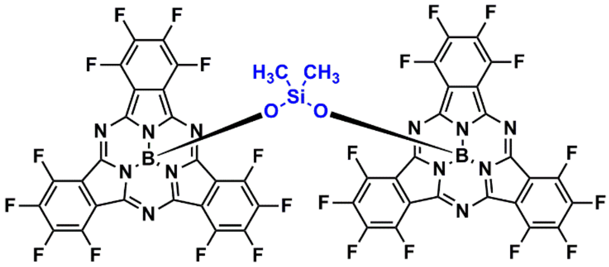

^{1}H-NMR (300 MHz, $CDCl_3$, 298 K): δ (ppm) = -1.71(s, 6H)

APCI-HRMS ($C_{50}H_7B_2F_{24}N_{12}O_2Si$) $[M+H]^+$: Calculated: 1313.0398; Found: 1313.0390

UV-Vis (Toluene): λmax (nm) (log ε) = 568 (4.5), 556 (sh), 531 (sh), 302 (4.5)

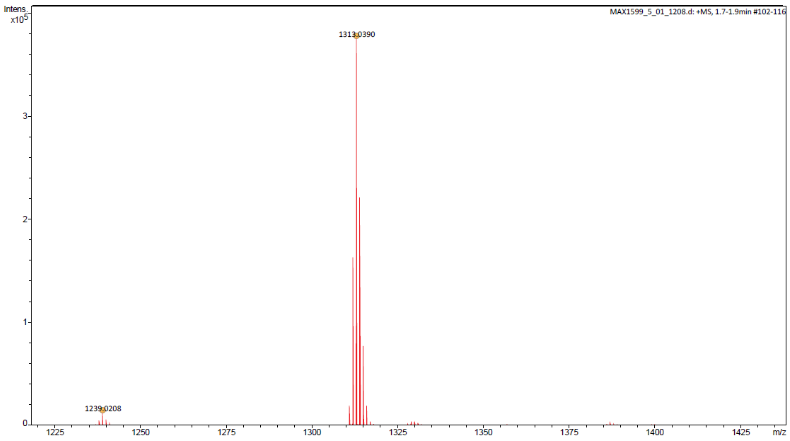

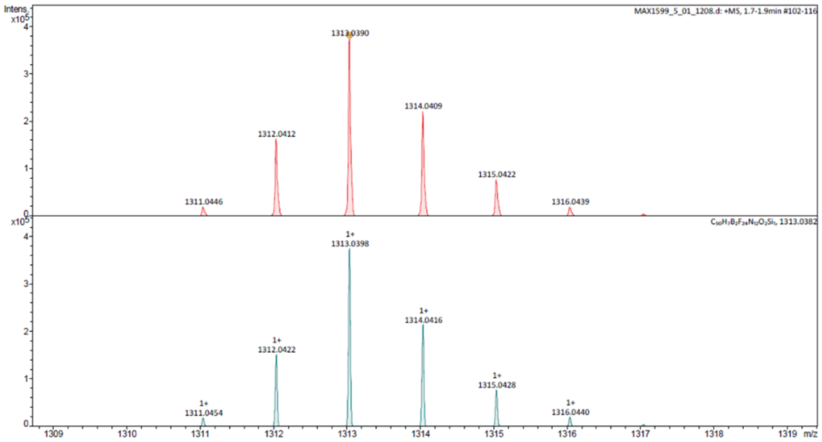

Figure S20. APCI spectra of SubPc **6**

1.3. NMR and mass spectra of Subphthalocyanines 3a-3h

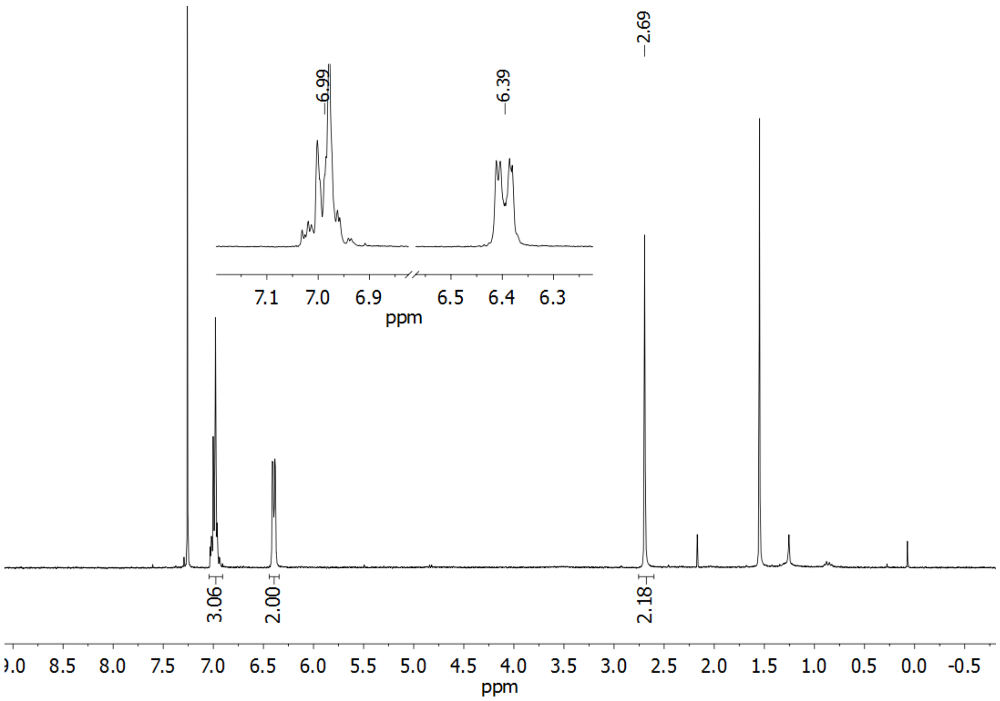

Figure S21. ^{1}H-NMR of **3a** in $CDCl_3$ (r.t).

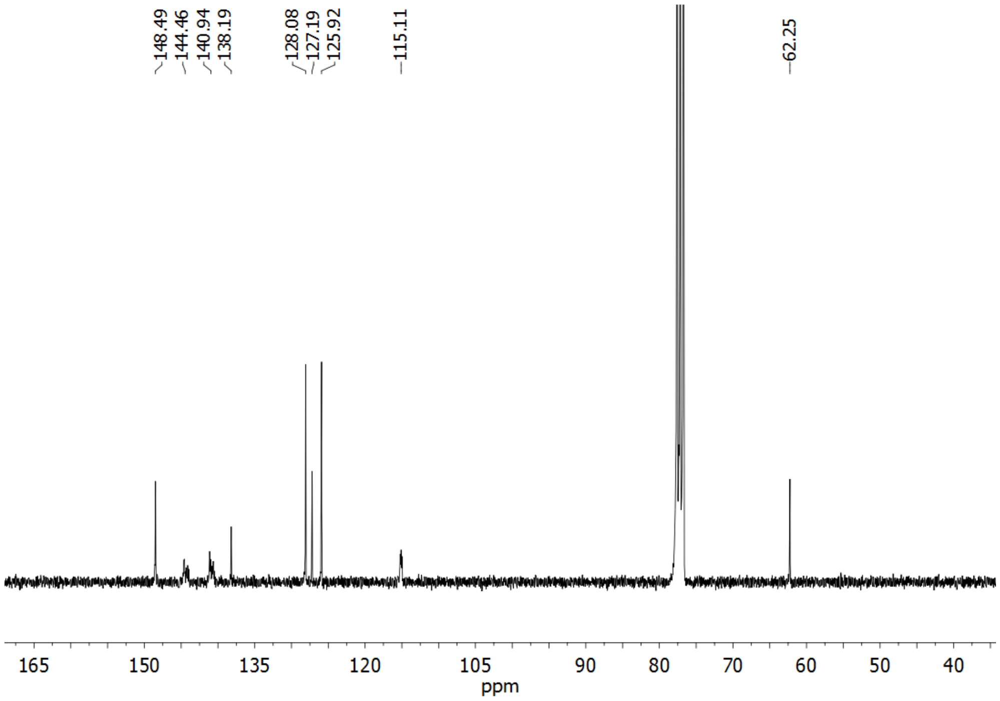

Figure S22. ^{13}C-NMR of **3a** in $CDCl_3$ (r.t).

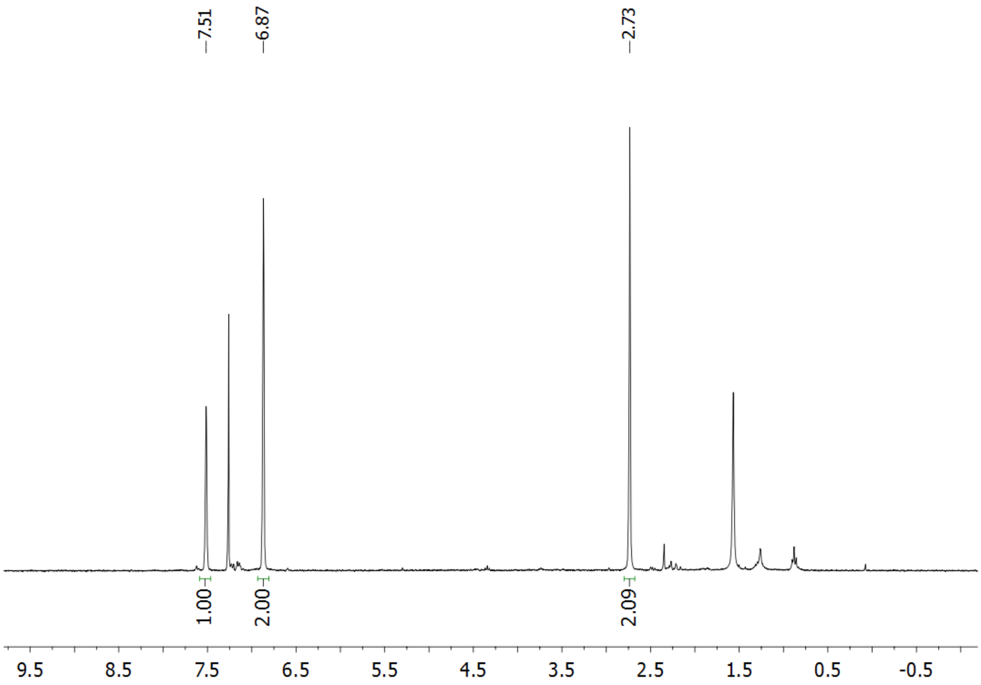

Figure S23. ^{1}H-NMR of **3b** in $CDCl_3$ (r.t).

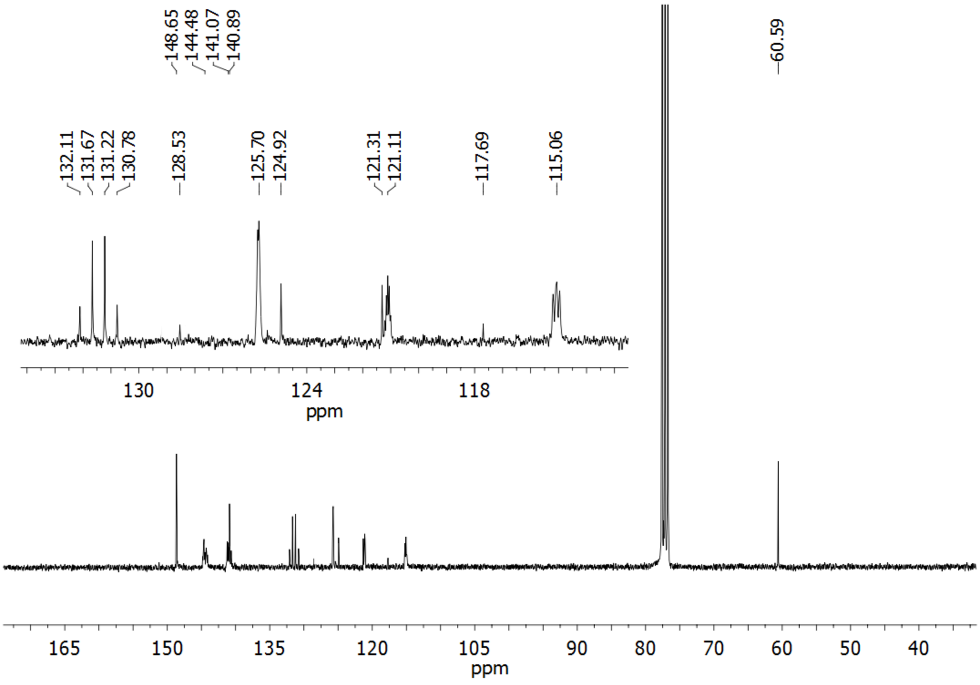

Figure S24. ^{13}C-NMR of **3b** in $CDCl_3$ (r.t).

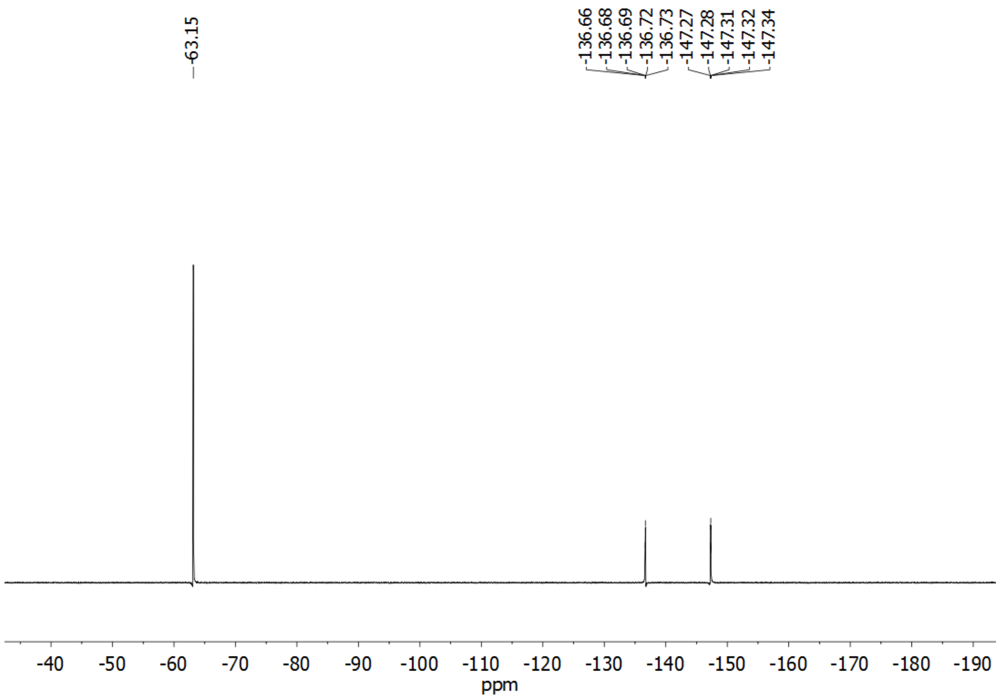

Figure S25. ^{19}F-NMR of **3b** in $CDCl_3$ (r.t).

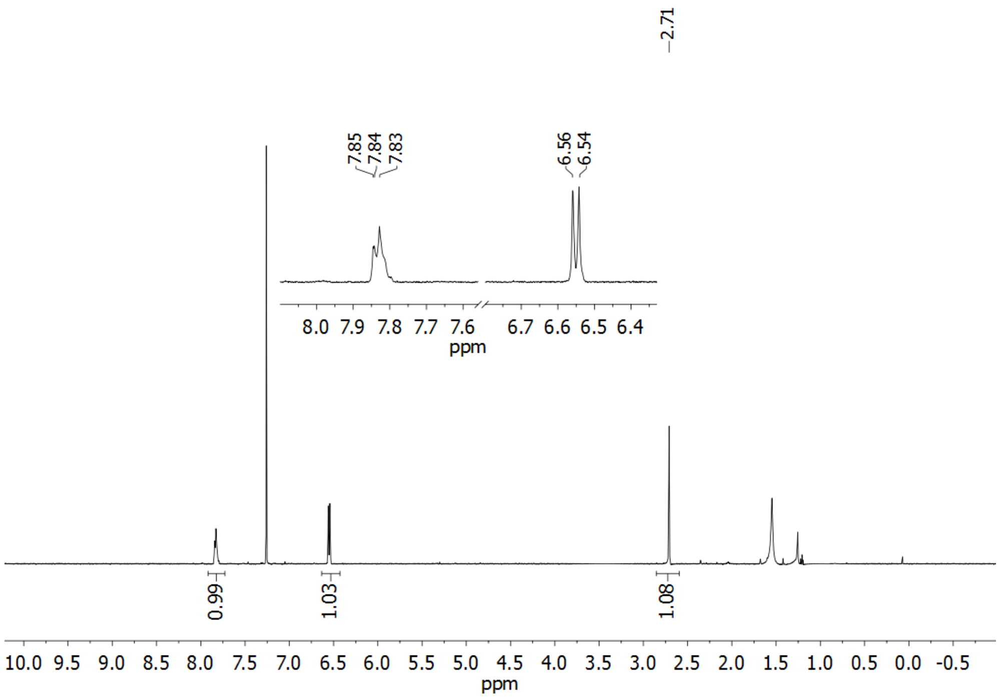

Figure S26. ^{1}H-NMR of **3c** in $CDCl_3$ (r.t).

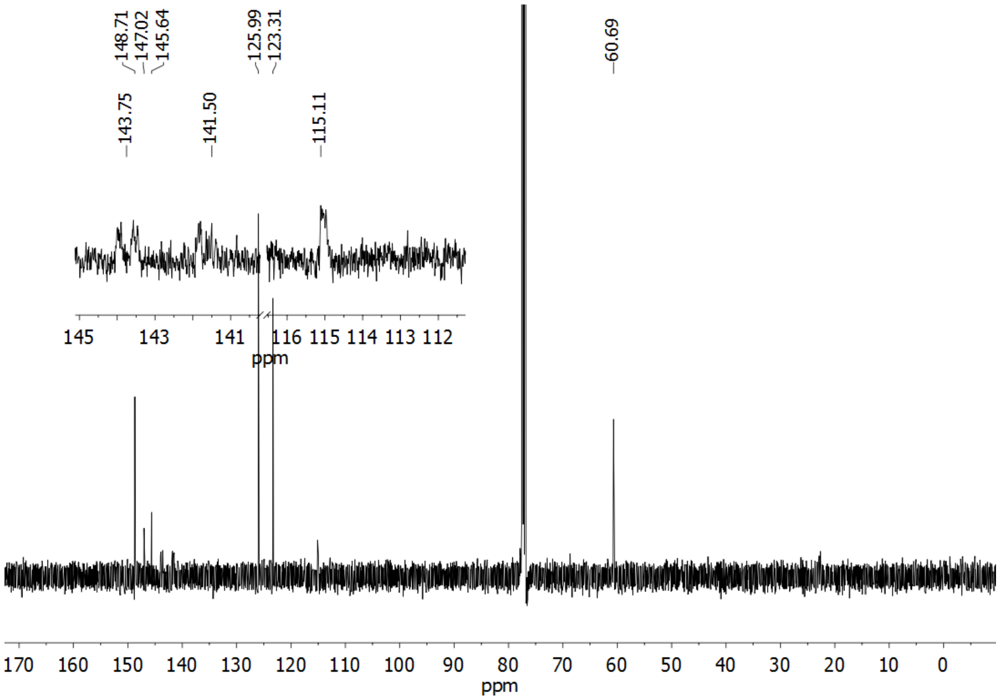

Figure S27. ^{13}C-NMR of **3c** in $CDCl_3$ (r.t)

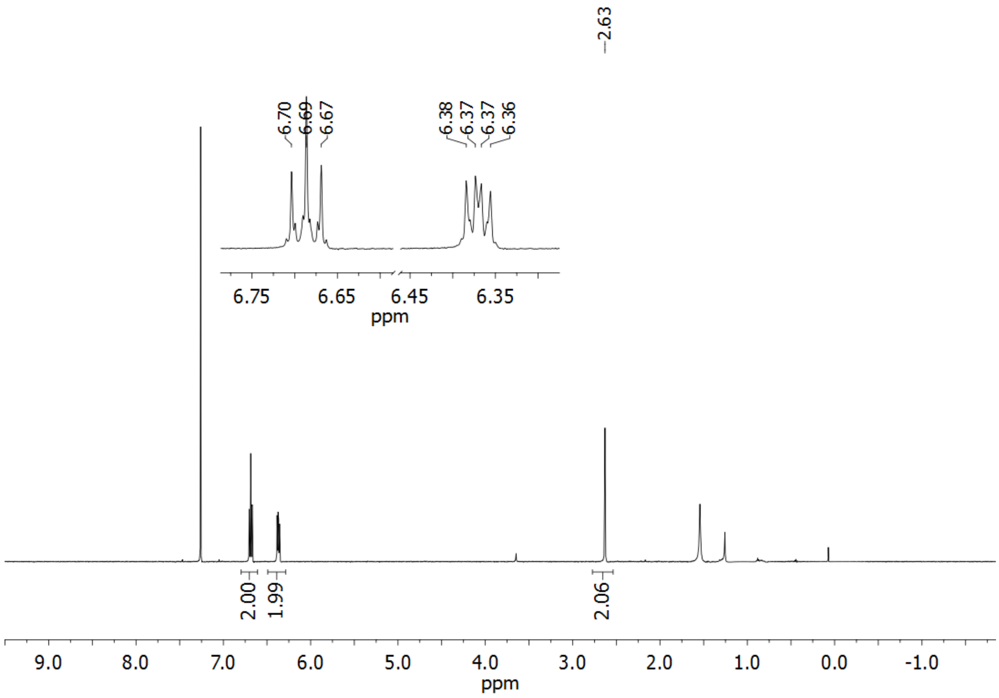

Figure S28. ^{1}H-NMR of **3d** in $CDCl_3$ (r.t).

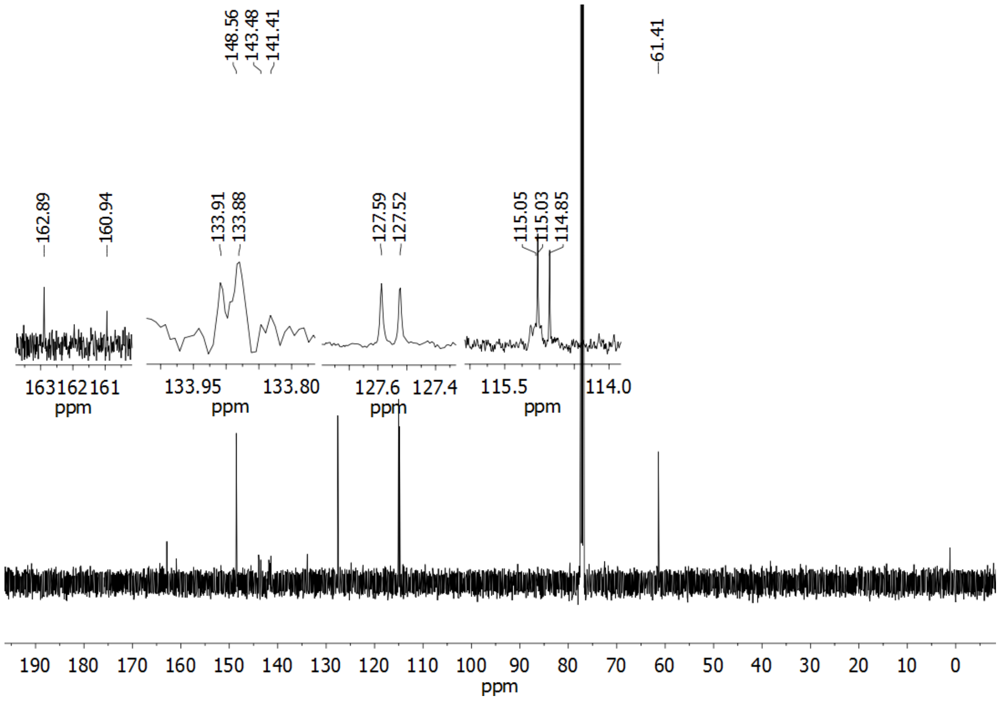

Figure S29. ^{13}C-NMR of **3d** in $CDCl_3$ (r.t).

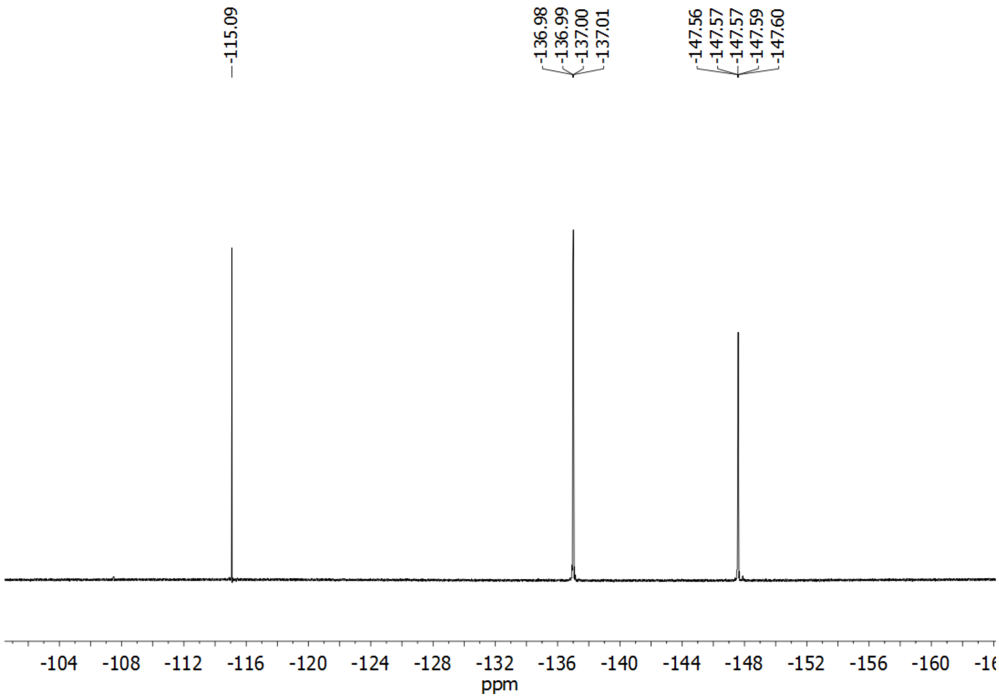

Figure S30. ^{19}F-NMR of **3b** in $CDCl_3$ (r.t).

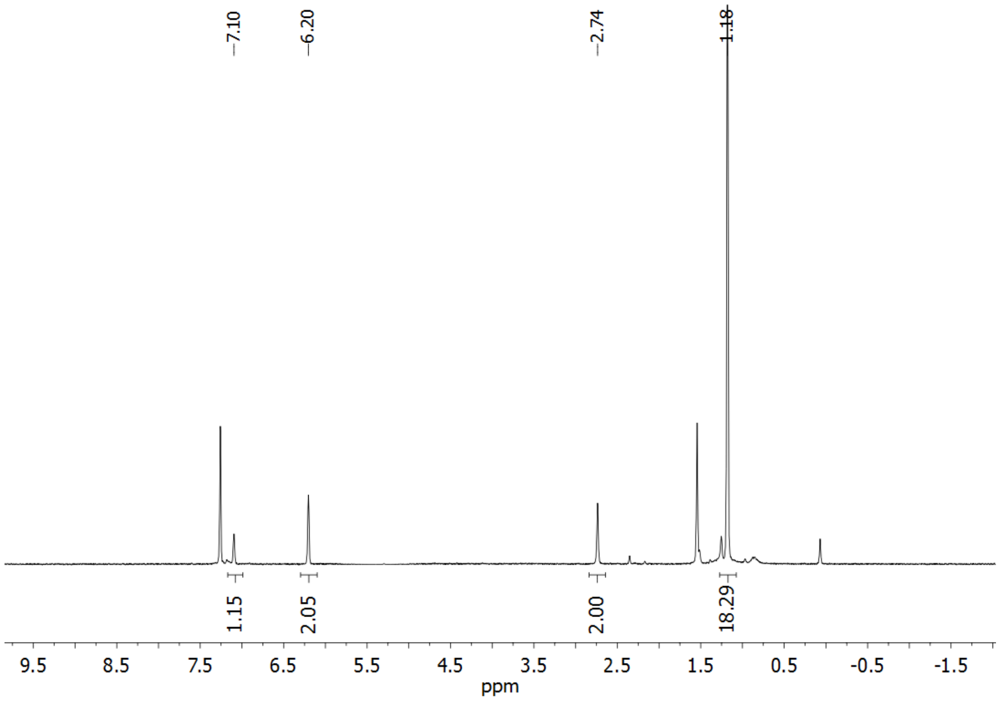

Figure S31. ^{1}H-NMR of **3e** in $CDCl_3$ (r.t).

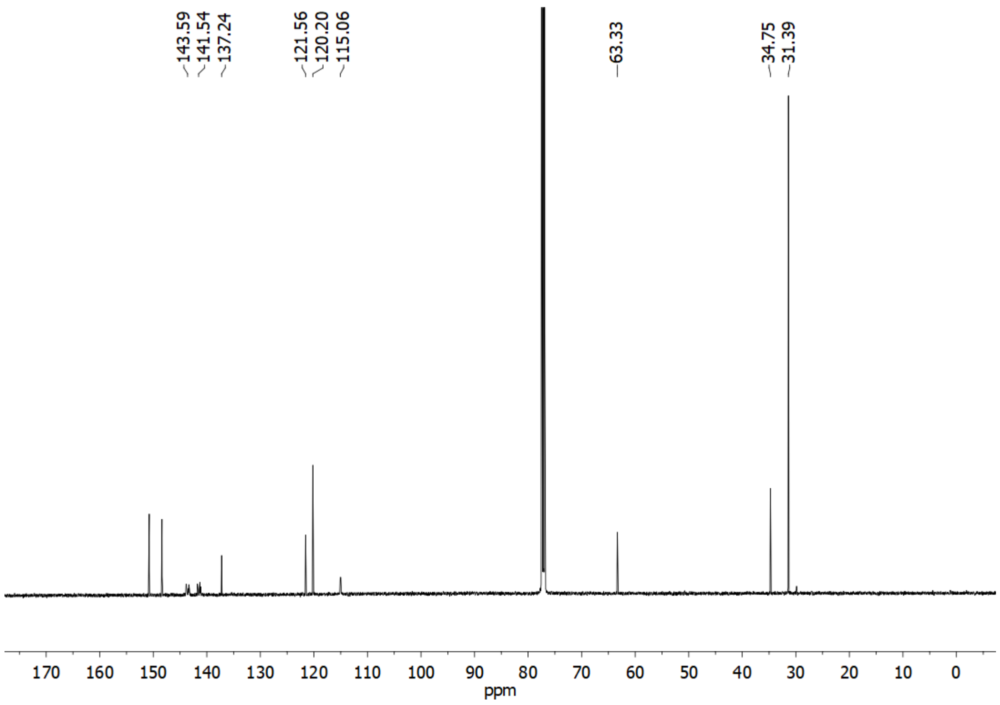

Figure S32. ^{13}C-NMR of **3e** in $CDCl_3$ (r.t).

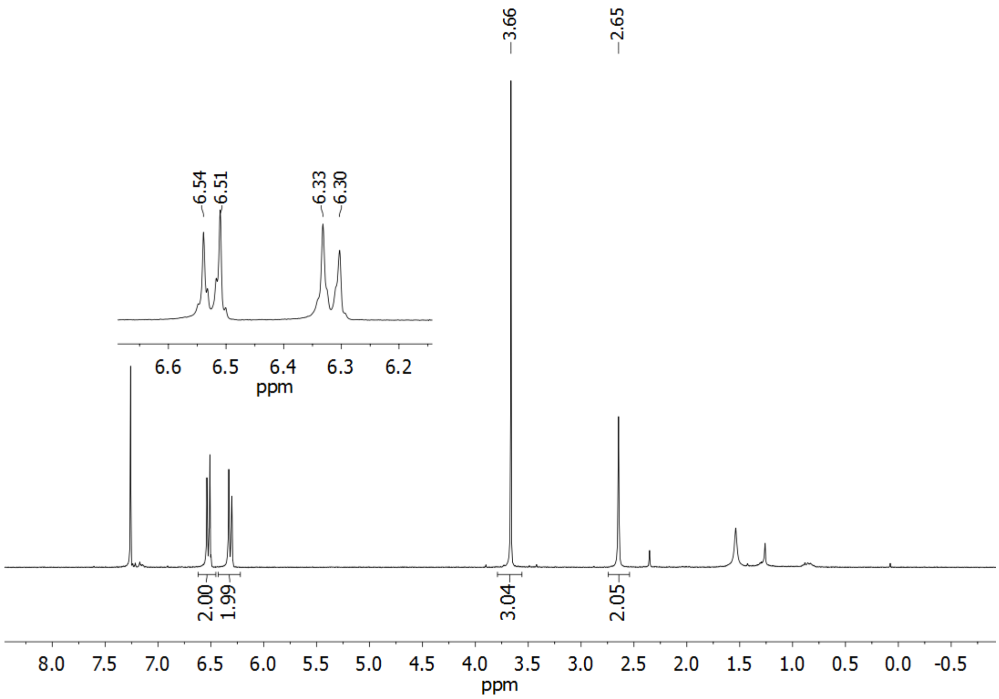

Figure S33. ^{1}H-NMR of **3f** in $CDCl_3$ (r.t).

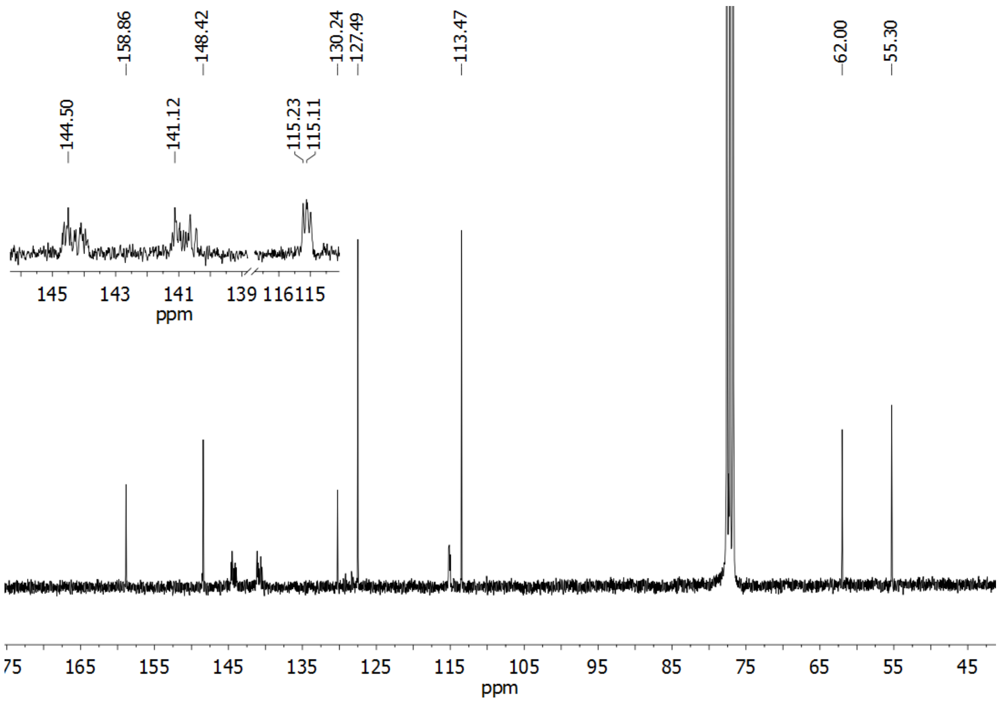

Figure S34. ^{13}C-NMR of **3f** in $CDCl_3$ (r.t).

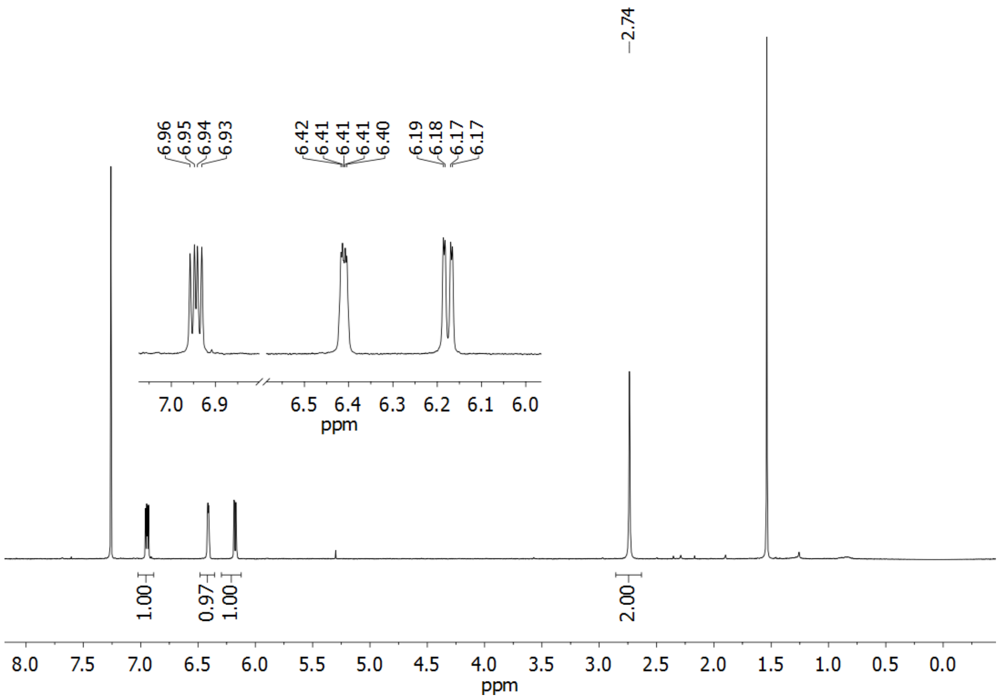

Figure S35. ^{1}H-NMR of **3g** in $CDCl_3$ (r.t).

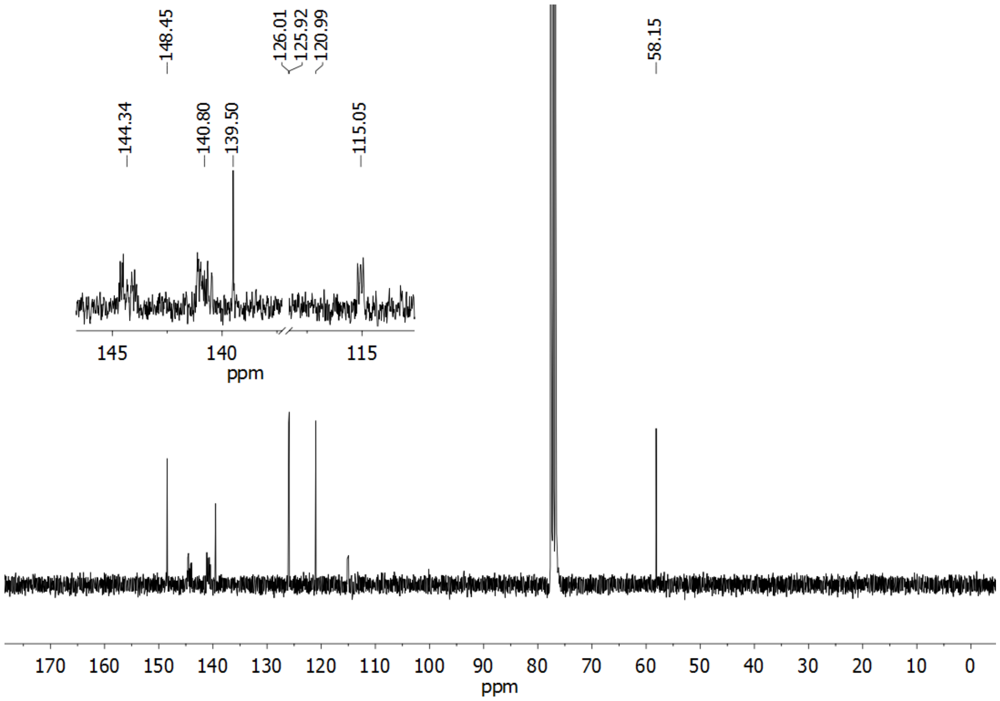

Figure S36. ^{13}C-NMR of **3g** in $CDCl_3$ (r.t).

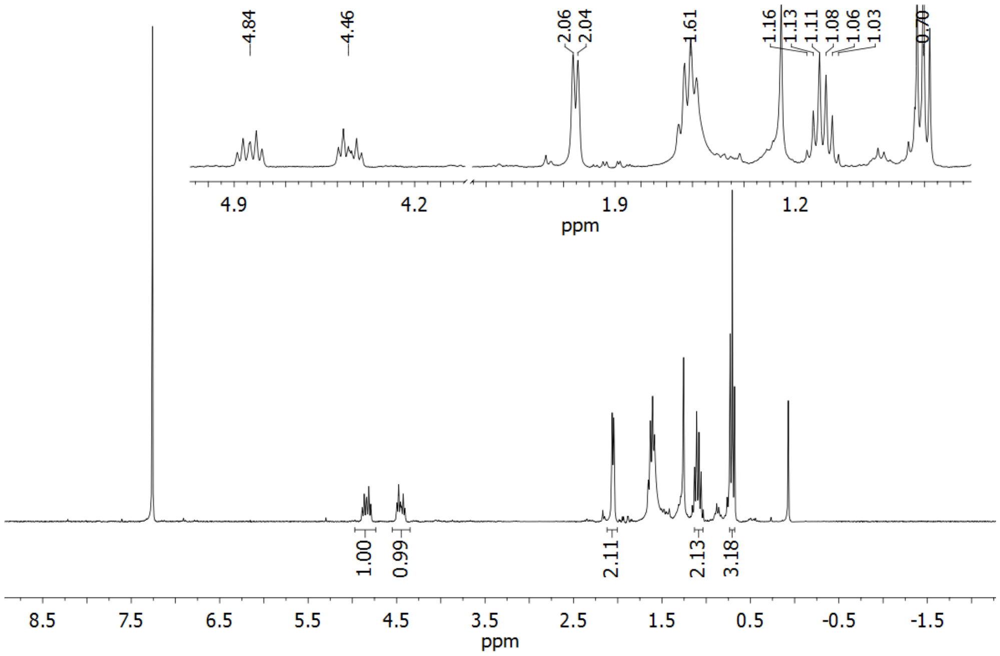

Figure S37. ^{1}H-NMR of **3h** in $CDCl_3$ (r.t).

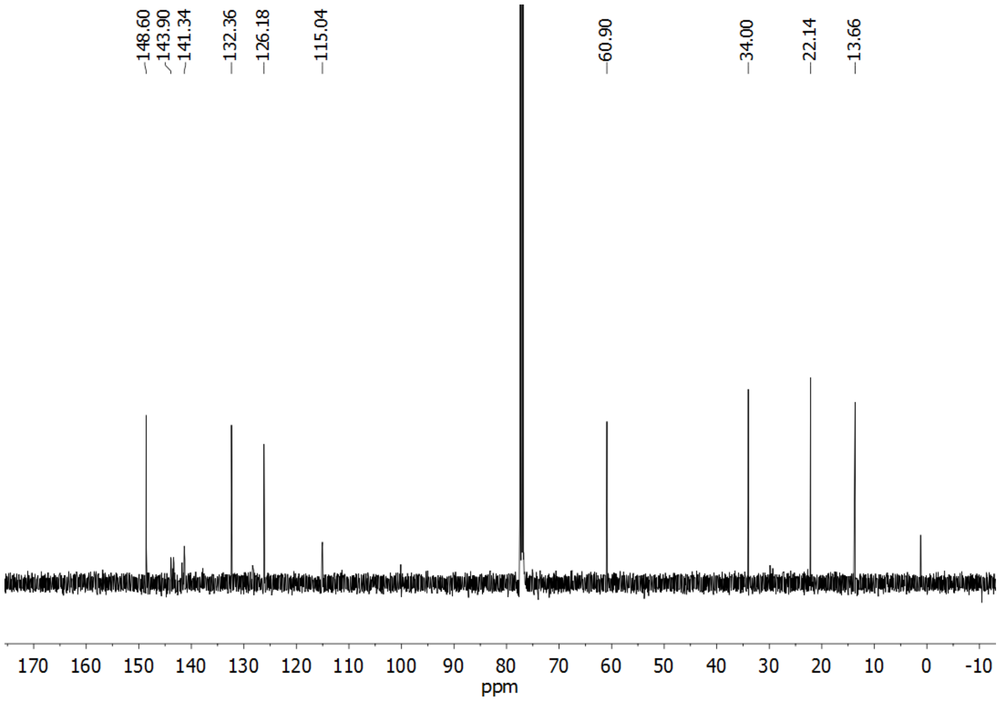

Figure S38. ^{13}C-NMR of **3h** in $CDCl_3$ (r.t).

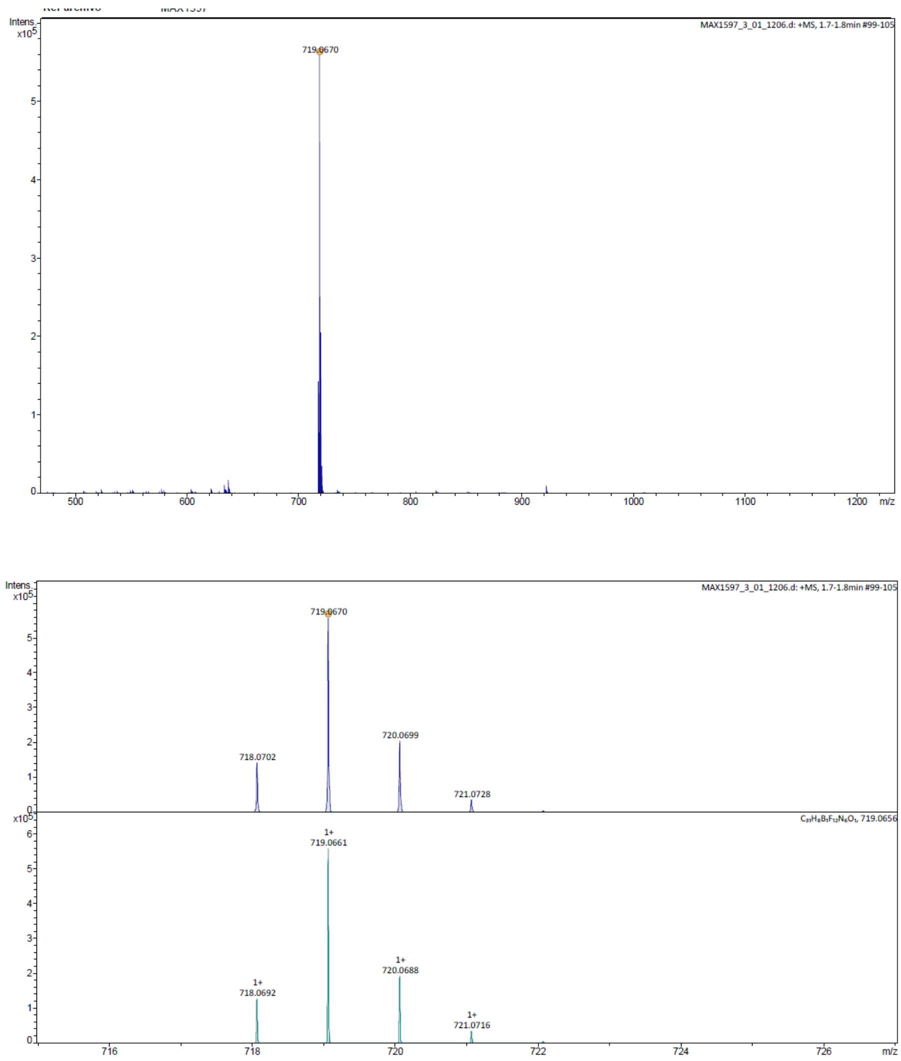

Figure S39. APCI-HRMS of **3a**

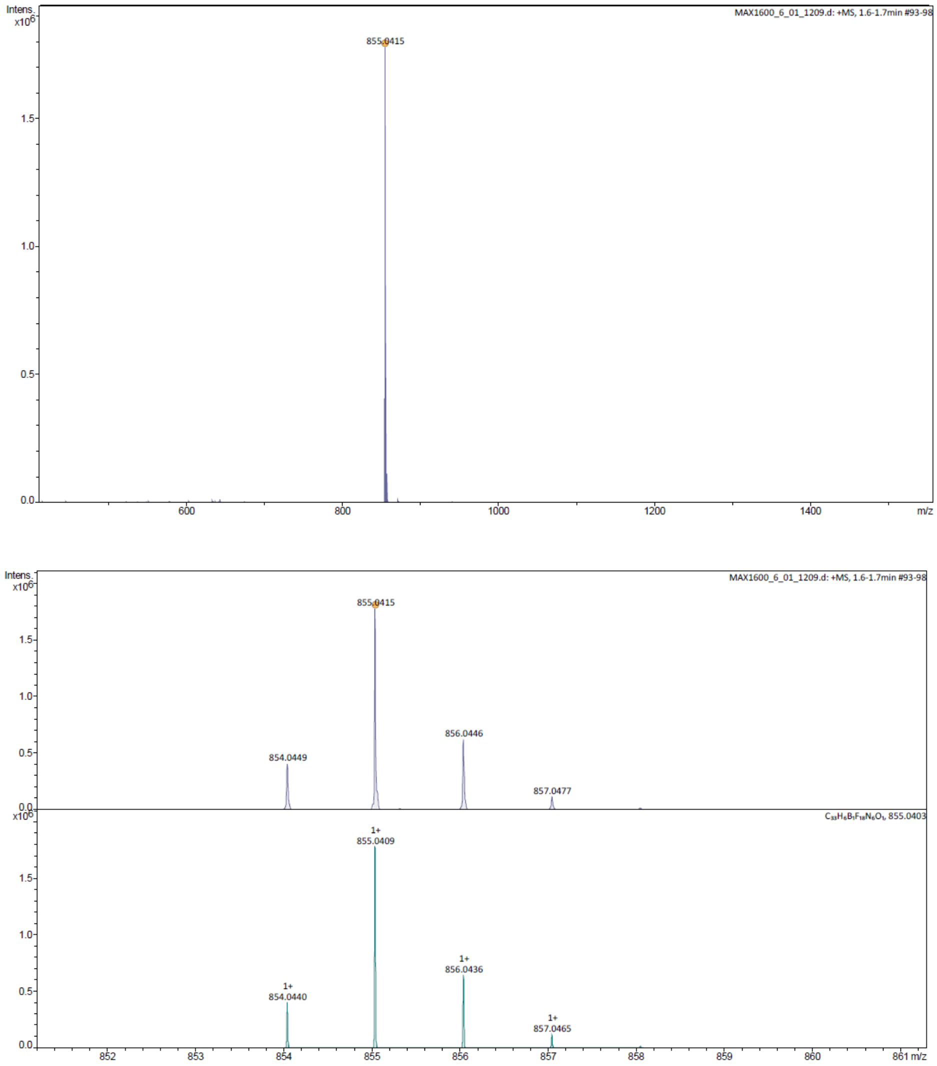

Figure S40. APCI-HRMS of **3b**

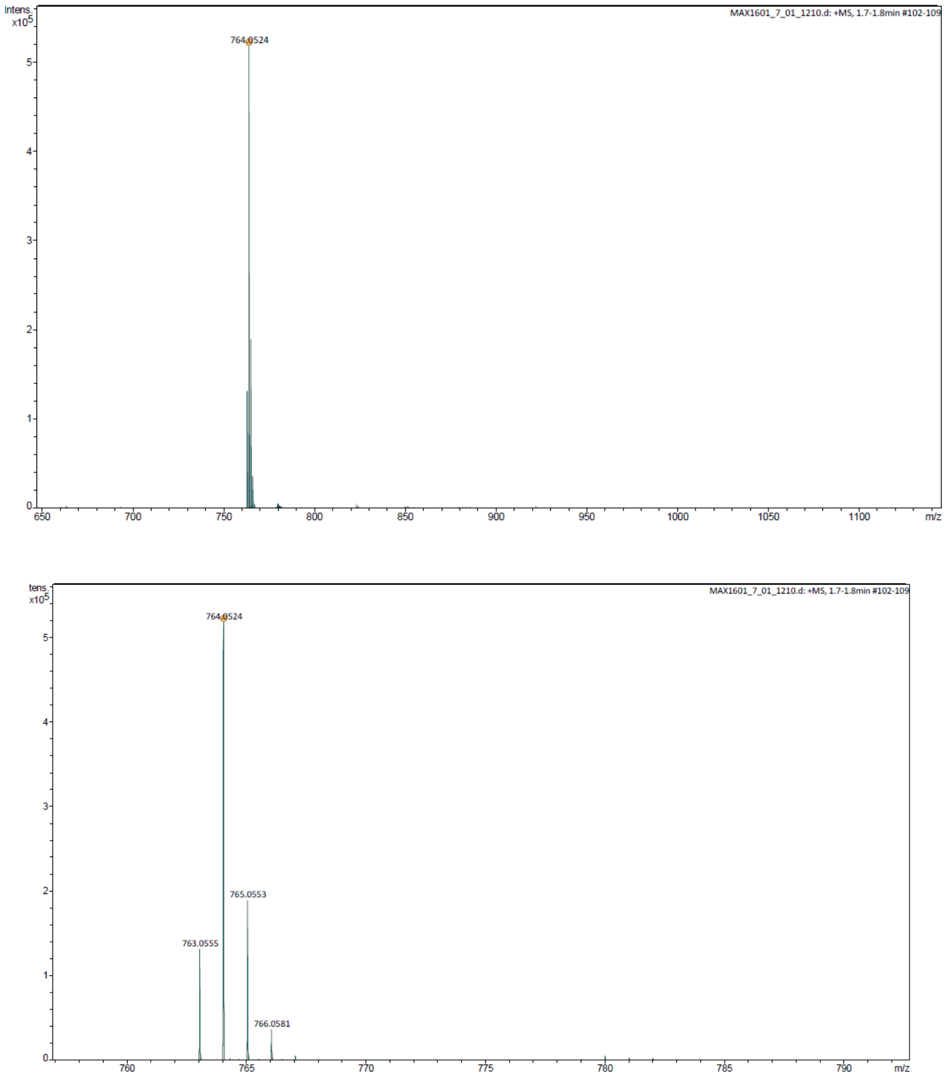

Figure S41. APCI-HRMS of **3c**

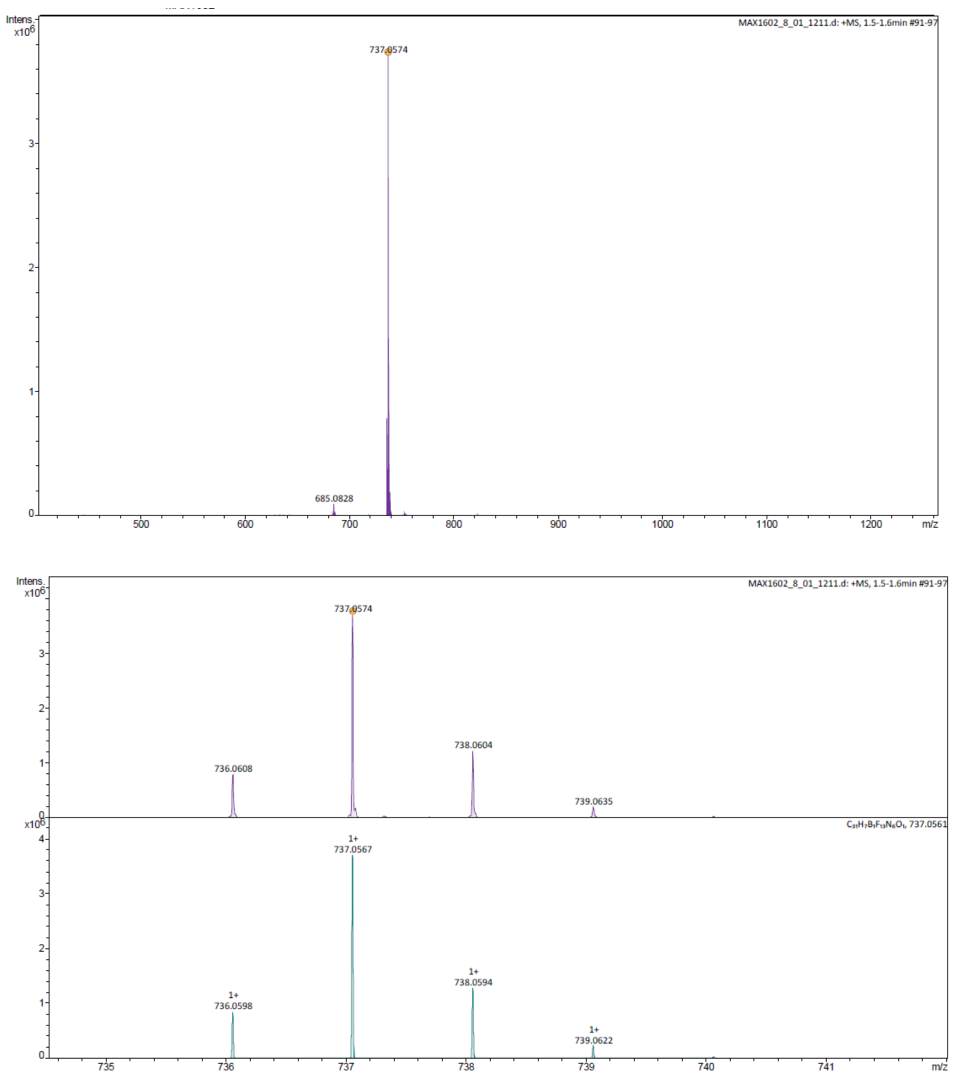

Figure S42. APCI-HRMS of **3d**

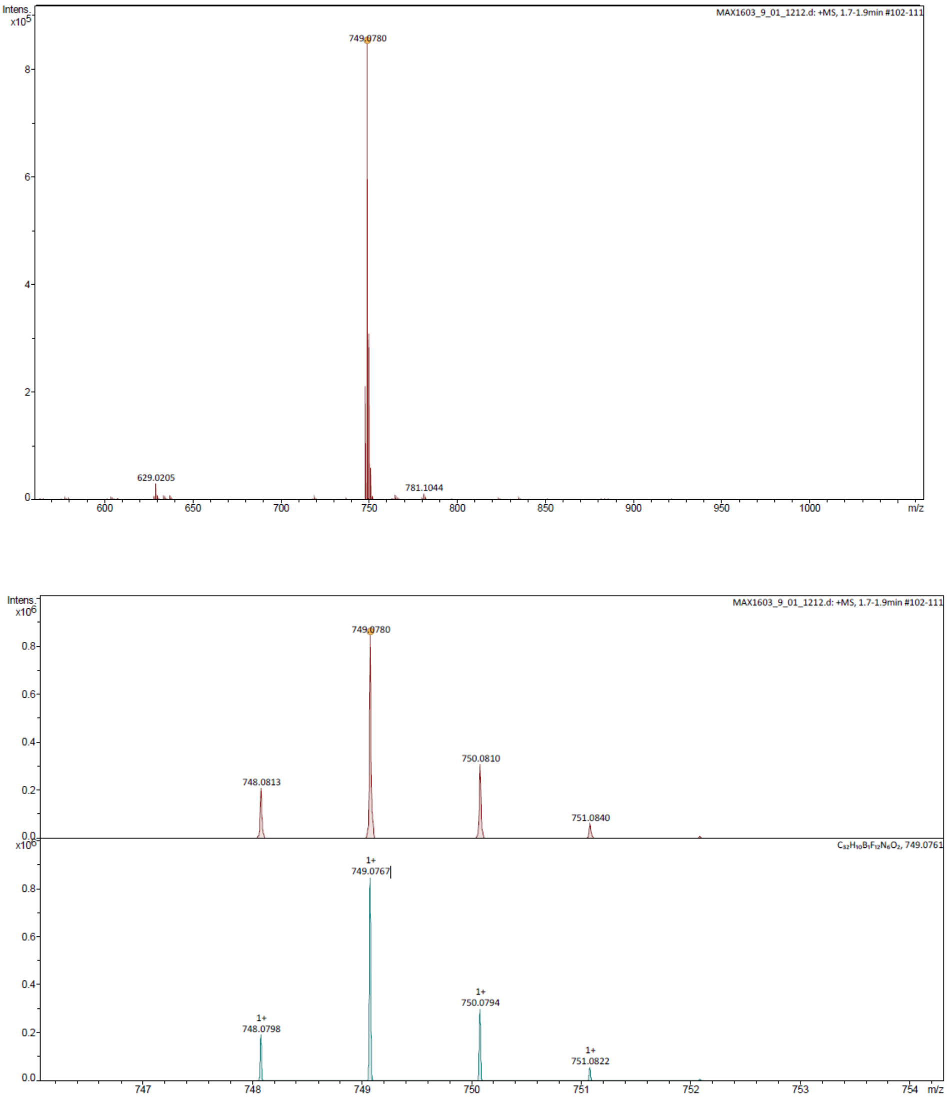

Figure S43. APCI-HRMS of **3f**

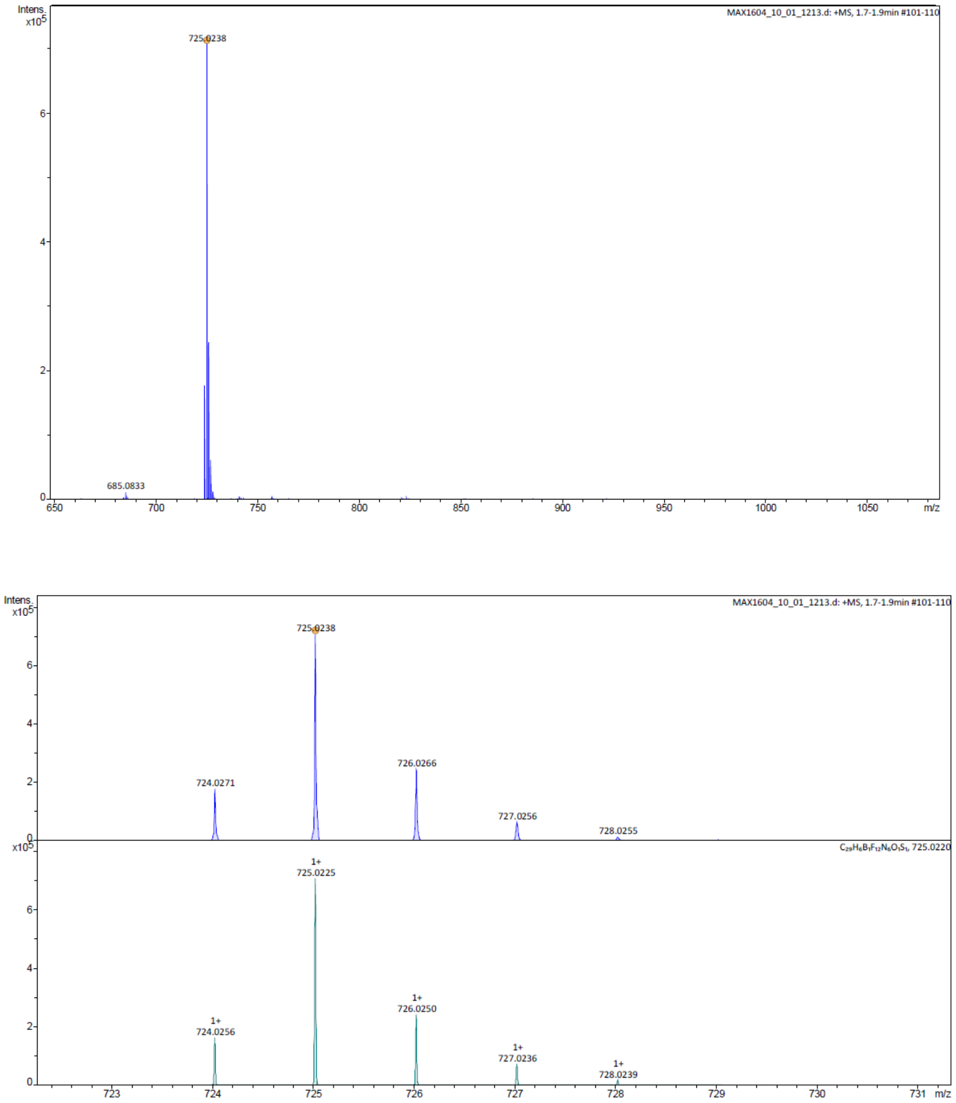

Figure S44. APCI-HRMS of **3g**

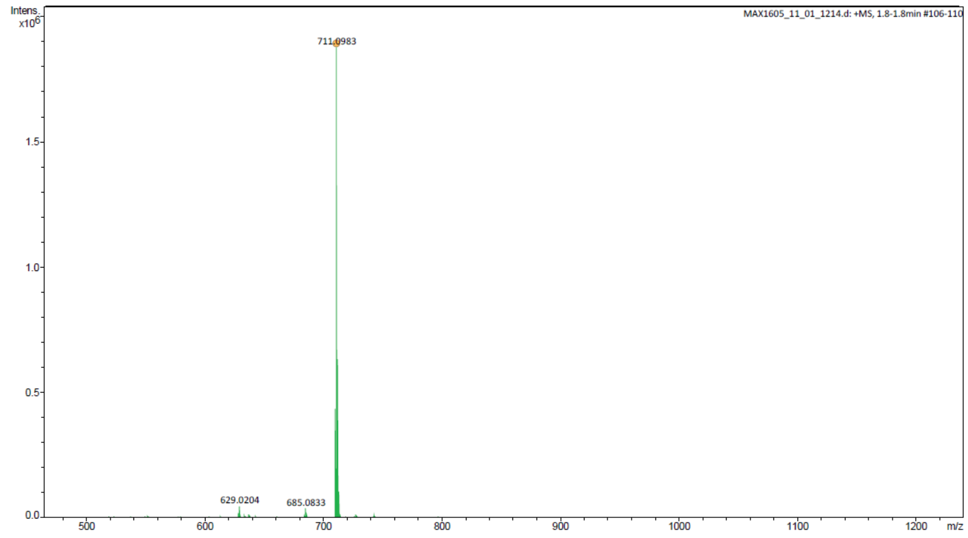

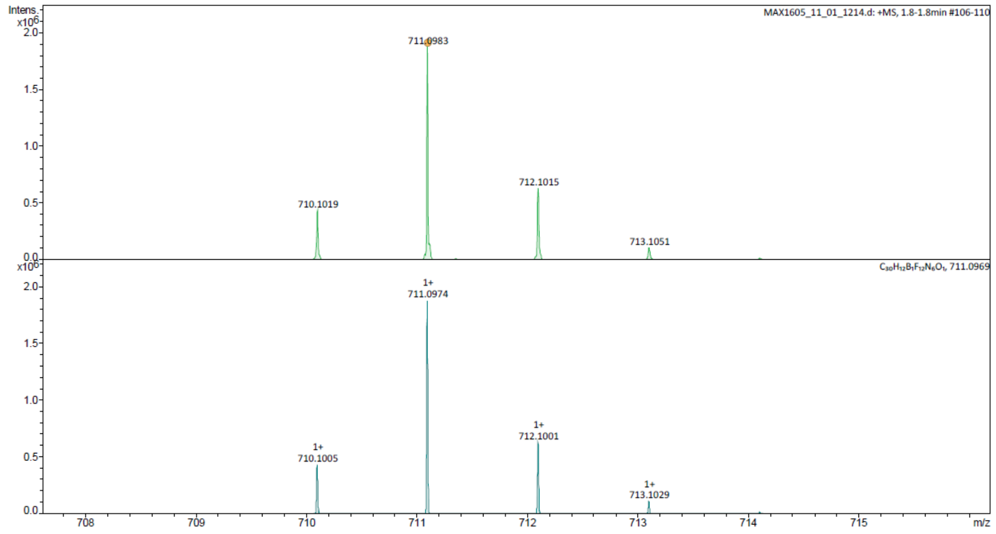

Figure S45. APCI-HRMS of **3h**

1.4. Reaction Mechanism considerations

1.4.1. Borenium catalysis

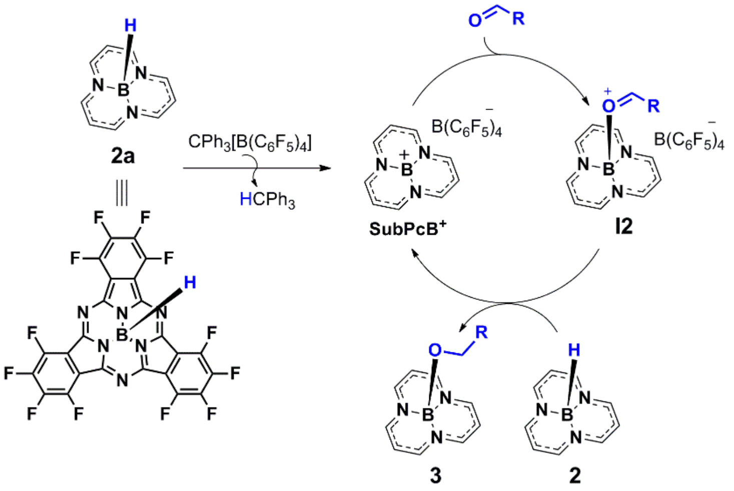

Scheme S2. Plausible mechanism for hydroboration of aldehydes under Borenium catalysis.[7]

The reaction would be initiated by abstraction of the hydride of **2a** by the trityl cation to generate the SubPc Borenium cation **(SubPcB$^+$)**, which would activate the aldehyde to form the cationic intermediate **I2**. Subsequent hydride transfer from **2a** to **I2** would give the product **3** and the regeneration of the catalyst.

1.4.2. TMSOTf Catalysis:

1.4.2.1. Discarded Mechanistical proposal and transmetalation step experiment.

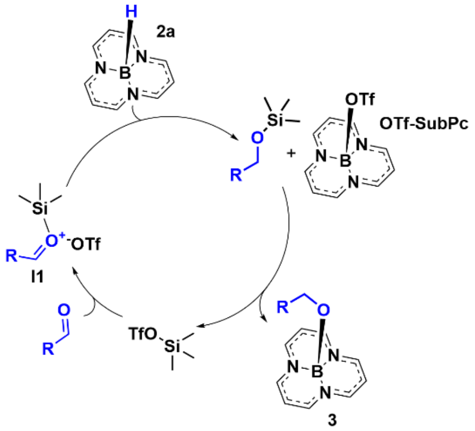

Scheme S3. Plausible mechanism for hydroboration of aldehydes under TMSOTf catalysis.

[7] Similar proposal to the previous one reported by Osuka (E. Tsurumaki, J. Sung, D. Kim, A. Osuka, *J. Am. Chem. Soc.* **2015**, *137*, 1056-1059).

The reaction would be initiated by the coordination of the aldehyde to TMSOTf and then the hydride transfer from **2a** to **I1** would take place originating the corresponding silyl ether and **OTf-SubPc**.[8] Then, a transmetalation between **OTf-SubPc** and the silyl ether would yield **3** and the regeneration of TMSOTf.

1.4.2.2. Experiment to study the feasibility of the transmetalation step:

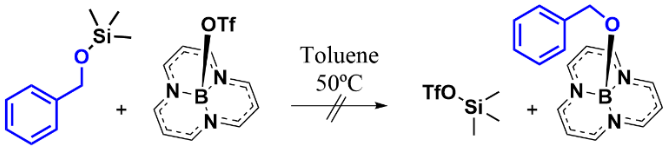

OTf-SubPc **3a**

SubPc **4** (13.5 μmol) and trimethylsilyl benzyl ether (13.7 μmol) were dissolved in dry toluene (1 ml) under argon atmosphere. The reaction mixture was stirred at 60 °C and the reaction progress monitored by TLC. After 24h, no consumption of **4** was detected.[9]

2. Computational study

All the stationary points were optimized in the frame of Density Functional Theory (DFT), using the wB97X-D[10] functional and the def2svp[11] basis set. This functional was selected due to its good performance for treating similar systems[12] in part due to the inclusion of empirical dispersion, and also analogous reaction mechanisms. For each optimized structure, analytic harmonic frequencies were computed at the same level of theory to confirm the nature of the stationary points. Furthermore, transition states were connected with reactants and products, when possible, via Intrinsic Reaction Coordinate (IRC) calculations. Solvent (toluene) effects were also taken into account through the implicit Polarizable Continuum Model (PCM).[13]

All the calculations were performed using the Gaussian09 program.[14]

[8] Resulting from the bonding between the triflate anion and the SubPcB$^+$ cation generated after hydride transfer.
[9] The experiment suggests that **4** is not formed, otherwise the reaction would stop and **4** would be the reaction product.
[10] J. Chai, M. Head-Gordon, *J. Chem. Phys*. **2008**, *128*, 084106.
[11] a) F. Weigend, *Phys. Chem. Chem. Phys*. **2006**, *8*, 1057-1065. b) F. Weigend, R. Ahlrichs, *Phys. Chem. Chem. Phys*. **2005**, *7*, 3297.
[12] a) Z. Chen, W. Yu, *Mol. Phys*. **2017**, *115*, 424. b) M. E. El-Khouly, C. Goel, M. M. El-Hendawy, S. Yesilot, M. Durmus, *J. Porphyrins Phthalocyanines* **2015**, *19*, 261.
[13] a) J. Tomasi, B. Mennucci, R. Cammi, *Chem. Rev*. **2005**, *105*, 2999. b) S. Miertus, E. Scrocco, J. Tomasi, *Chem. Phys*. **1981**, *55*, 117.
[14] M. J. Frisch *et al.* Gaussian 09, revision A.02. (Gaussian Inc., 2009).

2.1. Aldehyde coordination mechanism

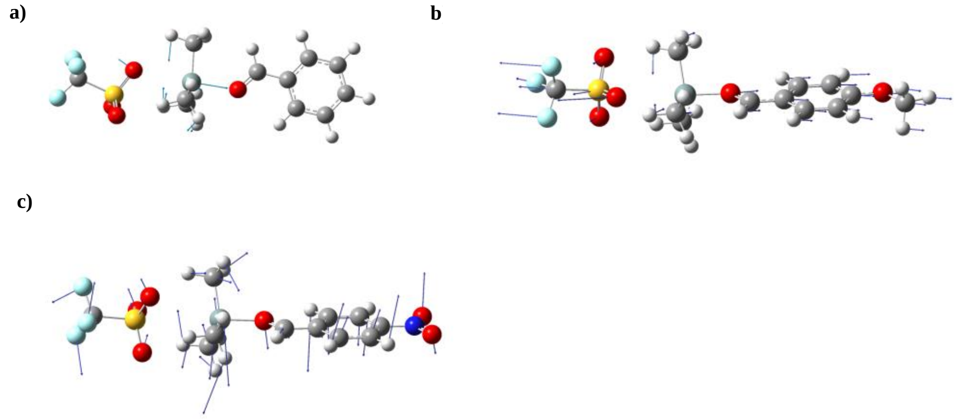

Figure S46. Displacement vectors associated to the imaginary frequencies characterizing the optimized transition states for the different benzaldehydes a) H (aldehyde **a**), b) -OMe (aldehyde **f**), and c) NO_2 (aldehyde **c**). Atom´s colour code: oxygen in red, fluorine in cyan, sulphur in yellow, carbon in grey, hydrogen in white, nitrogen in dark blue and silicon in teal

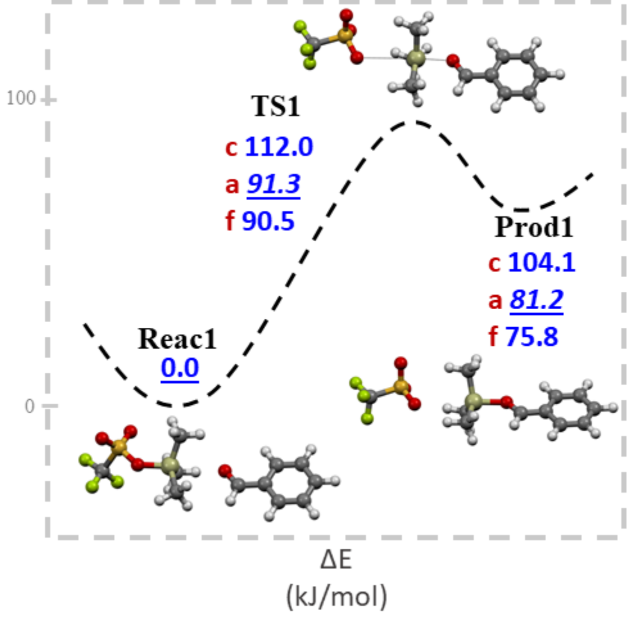

Figure S47. Potential energy profile for the activation of the aldehyde with TMSOTf. Atom´s colour code: carbon in grey, fluorine in lime, hydrogen in white, oxygen in red, silicon in pale green and sulphur in yellow

2.2. Hydride transfer mechanisms

2.2.1. Reactants

Reverse IRC from the **3mTS** ends in a stationary point in which both reactants are oriented along our reaction coordinate. However, due to the large size of our system, and its flexibility for instance on the relative orientation of the aldehyde and the SubPc, we have checked that our considered reaction coordinate is the most favoured path in solution. Relaxed scan along the Φ dihedral (Figure S48) and the C-H distance confirmed that: 1) our reactants, where the aldehyde

is stacked with the SubPc (Φ~170°) and with a distance ~3 Å , are the most stable arrangement, 2) they are connected to the products through a TS characterized by a ~1.7 Å C-H distance and further revealed that 3) if the aldehyde is displaced towards more perpendicular arrangement (Φ~100°) the energy barrier between reactants and products decreases, but this rotation also requires some energy.

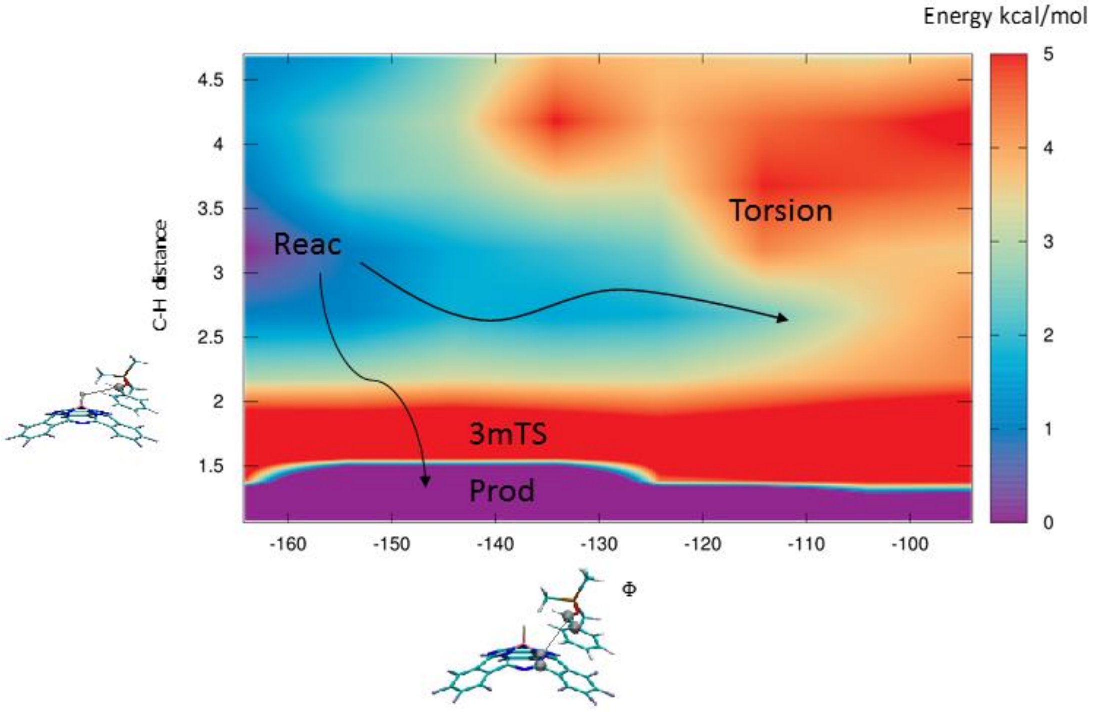

Figure S48. 2D Potential Energy Surface along the Φ dihedral (atoms involved in the dihedral are highlighted with grey spheres, the rest of the system depicted in sticks) and C-H distance

2.2.2. Transition states

2.2.2.1. Mechanism through the constrained 4 membered TS, **4mTS^c^**

The geometry of **4mTS**^{c} was located through constrained optimization where the B-O (2Å) and the B-H (1.7Å) distances were kept frozen. Longer B-H or B-O distances led to other transition states, none of them corresponding to the hydride transfer process.

The resulting optimized geometry is characterized by an imaginary frequency (-262 cm^{-1}) wherein the B-O bond and the B-H bond, respectively, form and break (Figure S49).

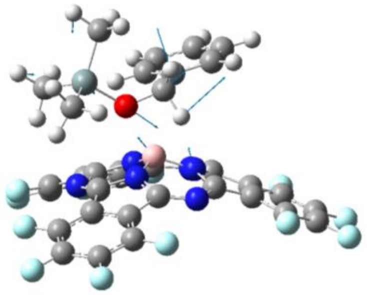

Figure S49. Displacement vectors associated to the imaginary frequency (-262 cm^{-1}) characterizing the optimized constrained geometry of **4mTS^{c}**

2.2.2.2. Mechanism through **3mTS**

The geometries for the optimized reactants and products are depicted in Figure S50a and c. The transition state **3mTS** (Figure S50b) is characterized by a single imaginary frequency (-336 cm^{-1}) wherein the B-H bond breaks simultaneously to the formation of the C-H bond. At the same time, the C-O bond distance increases, as expected due to the rehybridization of the C atom. The connection between this **3mTS** and reactants and products was confirmed performing IRC calculations.

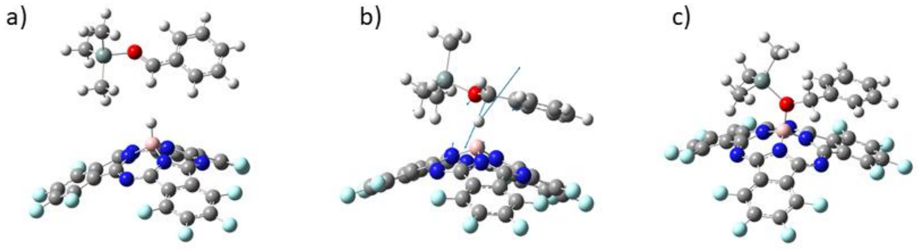

Figure S50. a) Reagents, b) Displacement vectors associated to the imaginary frequency (-336 cm^{-1}) characterizing the optimized constrained geometry of **3mTS**, c) product

2.2.2.3. Isodensity surface colour-coded with the Electrostatic Potential (ESP).

To get further insight on the larger stability of **3mTS** with respect to **4mTS^{c}**, we have computed at the corresponding geometries the associated isodensity surface colour-coded with ESP and charges. As inferred from Figure S51, the boron atom in the **4mTS^{c}** has a significantly higher (~2.5 fold) positive charge compared to that of **3mTS**, that would be stabilized by the presence of neighbour electron-rich atoms. However, the difference of the oxygen ESP charge between both TSs is small (+18%), because of its coordination to the trimethylsilyl fragment, suggesting that is not enough to achieve the boron-stabilization. At this point, the lowest energy pathway is, first, the formation of C-H thus the oxygen becomes more electron-rich and then, the formation of the B-O bond. In addition, we also believe that the steric hindrance provided by the trimethylsilyl group could hinder the stabilization of **4mTS^{c}**.

To further support this hypothesis, the optimization of **3mTS** and **4mTS** without trimethylsilyl fragment, i.e. the benzaldehyde hydroboration without activation, were performed (**3mTS´**and **4mTS´**). Interestingly, unlike the previous case, only the four-member transition state **4mTS´** was located (Figure S51), being characterized by a single imaginary frequency where the B-O and C-

H bond are simultaneously formed. Contrary, attempts to optimize the **3mTS´** were unsuccessful even in the constrained optimization cases. The electrostatic potential map and charges of this **4mTS´** (Figure S52) show an even larger positive character of the Boron atom (1.848) but in this case the electronic density in the oxygen atom also increases significantly (+70%) compared with that of **4mTS^c**, stabilizing enough the interaction, in agreement with our hypothesis.

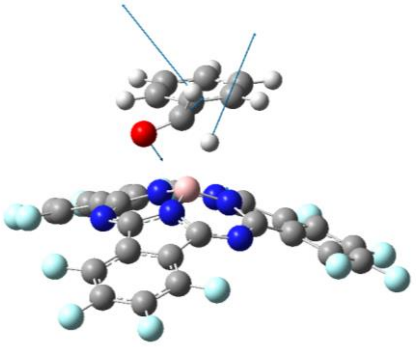

Figure S51. Displacement vectors associated to the imaginary frequency (-262 cm^{-1}) characterizing the optimized constrained geometry of **4mTS´**.

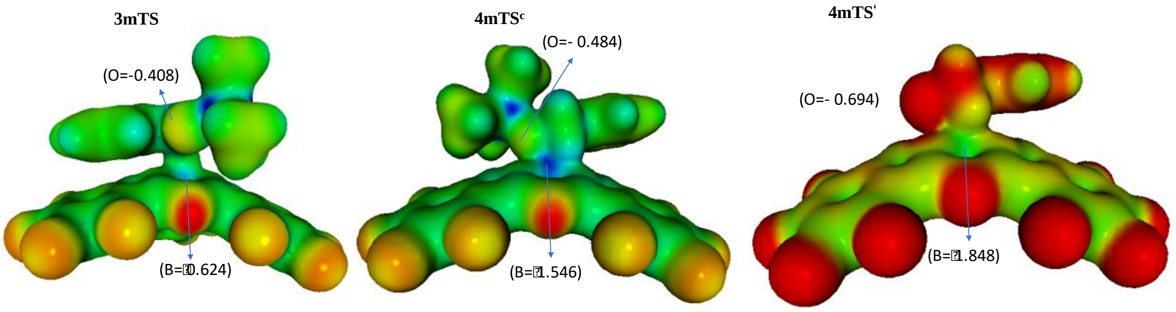

Figure S52. Electrostatic potential (and most representative ESP charges in a.u. in parenthesis) computed for the **3mTS** and **4mTS^c** and **4mTS’** transition states. ESP color code: red=0.05, yellow=0.1, green=0.2, light blue=0.3 and blue=0.4.
